# Lateral nanoarchitectonics from nano to life: ongoing challenges in interfacial chemical science

**DOI:** 10.1039/d4sc05575f

**Published:** 2024-10-28

**Authors:** Jingwen Song, Anna Jancik-Prochazkova, Kohsaku Kawakami, Katsuhiko Ariga

**Affiliations:** a Research Center for Functional Materials, National Institute for Materials Science (NIMS) 1-1 Namiki Tsukuba 305-0044 Ibaraki Japan; b Research Center for Materials Nanoarchitectonics, National Institute for Materials Science (NIMS) 1-1 Namiki Tsukuba 305-0044 Japan ARIGA.Katsuhiko@nims.go.jp; c Graduate School of Pure and Applied Sciences, University of Tsukuba 1-1-1 Tennodai Tsukuba 305-8577 Ibaraki Japan; d Graduate School of Frontier Sciences, The University of Tokyo 5-1-5 Kashiwa-no-ha Kashiwa 277-8561 Japan

## Abstract

Lateral nanoarchitectonics is a method of precisely designing functional materials from atoms, molecules, and nanomaterials (so-called nanounits) in two-dimensional (2D) space using knowledge of nanotechnology. Similar strategies can be seen in biological systems; in particular, biological membranes ingeniously arrange and organise functional units within a single layer of units to create powerful systems for photosynthesis or signal transduction and others. When our major lateral nanoarchitectural approaches such as layer-by-layer (LbL) assembly and Langmuir–Blodgett (LB) films are compared with biological membranes, one finds that lateral nanoarchitectonics has potential to become a powerful tool for designing advanced functional nanoscale systems; however, it is still rather not well-developed with a great deal of unexplored possibilities. Based on such a discussion, this review article examines the current status of lateral nanoarchitectonics from the perspective of in-plane functional structure organisation at different scales. These include the extension of functions at the molecular level by on-surface synthesis, monolayers at the air–water interface, 2D molecular patterning, supramolecular polymers, macroscopic manipulation and functionality of molecular machines, among others. In many systems, we have found that while the targets are very attractive, the research is still in its infancy, and many challenges remain. Therefore, it is important to look at the big picture from different perspectives in such a comprehensive review. This review article will provide such an opportunity and help us set a direction for lateral nanotechnology toward more advanced functional organization.

## Abstract 2

The construction of functional structures within a single plane still has a lot of challenges. This review article will help us find new groundbreaking directions in lateral nanoarchitectonics toward advanced functional material organization.

## Graphical abstract

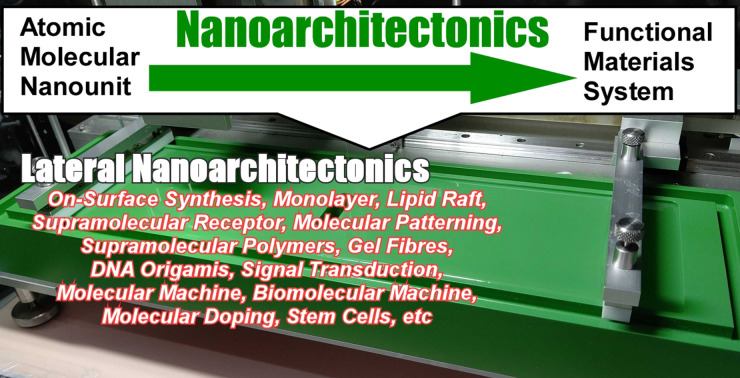

## 1. Introduction

The development of materials science is crucial for pushing the frontiers in modern society. Fabrication of advanced functional materials is of paramount importance when revolutionizing technologies that affect the daily life of humanity. Materials science is especially needed in developing sustainable energy production^1^ and storage systems^2^ (such as solar cells,^3^ fuel cells,^4^ batteries,^5^ and supercapacitors,^6^ among others), material conversion through catalysts,^7^ hydrogen production,^8^ sensors,^9^ devices,^10^ environmental purification technologies,^11^ and last but not least medical applications,^12^*i.e.* drug delivery,^13^ tissue engineering, *etc.*^14^

**Fig. 1:**
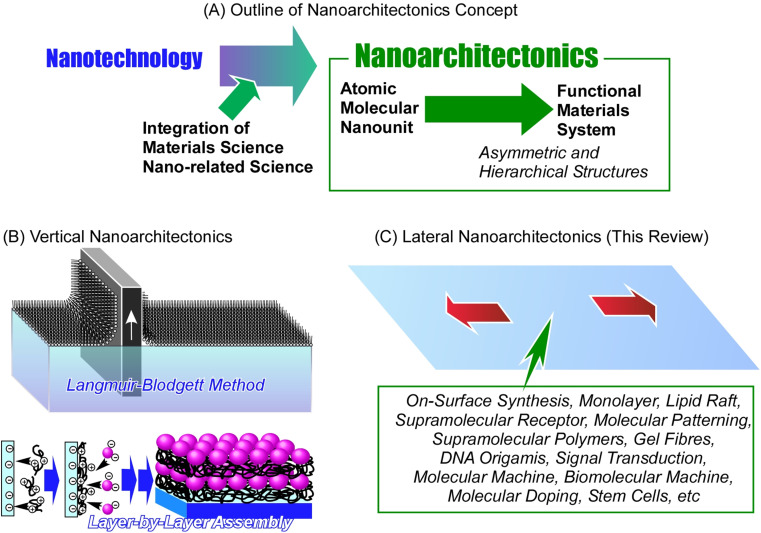
Nanoarchitectonics approach: (A) outline of the nanoarchitectonics concept; (B) vertical nanoarchitectonics for controlled layered organization carried out with well-known techniques such as the Langmuir–Blodgett film; (C) lateral nanoarchitectonics for controlled organization within the 2D plane as the main subject of this review discussed with a variety of objects.

Designing new functional materials is not limited only to chemical processes to fundamentally produce new materials with desired properties. Emphasis must be placed on the control of their supramolecular structure as well. During the long history of development, we have learned that it is extremely important to control not only the substance itself but also its nanostructure.^15^ This major trend has grown dramatically since Richard Feynman initiated the rise of nanotechnology in the middle of the 20th century.^16^ Nanotechnology enabled observation of nanoscale objects,^17^ creation of nanostructures,^18^ and evaluation of their physical properties.^19^ This technology has had a huge impact on the world of materials science. The development of functional materials was no longer possible without considering the final nanostructures; this gave rise to the great field of nanoarchitectonics – a post-nanotechnology successor to nanotechnologies.^20^ Nanoarchitectonics and its principles were first proposed by Masakazu Aono at the beginning of the 21st century^21^ as a method of designing functional materials from atomic and molecular nano-units with the knowledge of nanotechnology (Fig. 1).^22^ A similar concept is often used in self-assembly processes in supramolecular chemistry,^23^ the creation of metal–organic frameworks (MOF) in coordination chemistry,^24^ the preparation of covalent organic frameworks (COF) in polymer chemistry,^25^ and template synthesis in materials science.^26^ In terms of science and technology, it is desirable to treat these concepts as a unified field rising from general scientific pillars. Therefore, it can be said that nanoarchitectonics is not a novel approach but an incorporation of multiple concepts into one general field. In other words, nanoarchitectonics aims to integrate a wide range of disciplines within the nanofield.^27^ The general principles of nanoarchitectonics are not limited only to the field of material chemistry; they overlap with many other fields, such as microfabrication techniques, biochemical methods, and others, making it easy to design complex, asymmetric and hierarchical structures regardless of the chosen material or application.^28^ Not limited to non-covalent interactions commonly seen in supramolecular assembly, material construction in nanoarchitectonics approaches includes material expansions through covalent bonding. The concept of nanoarchitectonics is expanding from fundamental fields such as physics,^29^ chemistry,^30^ and biology^31^ to applied fields such as energy conversion,^32^ sensors,^33^ devices,^34^ and environmental,^35^ and biomedical applications.^36^ Since matter is fundamentally made up of atoms and molecules organized within space in a defined and organized way, nanoarchitectonics is a methodology that applies to all matter. If the ultimate goal of physics is to elucidate the theory of everything,^37^ analogously, the goal of nanoarchitectonics could be to achieve a method for everything in materials science.^38^

The method of skillfully organizing unit structures and assembling advanced functions is seen in living organisms. Functions such as photosynthesis and signal transduction are the result of the sophisticated arrangement and organization of functional units.^39^ The goal of nanoarchitectonics is to design these structures and material systems artificially.^40^ Biological systems have achieved the art of functional organization through trial-and-error learning over billions of years. We are trying to achieve this artificially in the last few decades, since postulating the principles of nanotechnologies. From this point of view, it is a very tough and complex challenge. Perhaps the first step should be to target the architecture of a local functional organization in 2D space, which is the main goal of lateral nanoarchitectonics. Lateral nanoarchitectonics assists in the design, fabrication, and characterization of functional 2D structures with defined properties on a macroscopic level. Precise control of the atomic and molecular organization is typically achieved at the interface environment.^41^ Architecting functional materials at the interface is a realistic yet simplified approach in a two-dimensional (2D) space. When we understand the general principles of nanoarchitectonics in such 2D systems, the path of organizing functions in a wide three-dimensional (3D) space will be clearer. Commonly used nanoarchitectonics methods applying interface science are the Langmuir–Blodgett (LB) method^42^ and layer-by-layer (LbL) assembly.^43^ These methods create skillfully defined layered structures and achieve functional coordination between them at the atomic and molecular levels.

Comparing the latter methodology with functional organization in biomembranes, a significant difference is observed. While the LB and LbL methods build layered functional structures, biomembranes sophisticatedly arrange and organize functional units within two-dimensional assemblies. This is a more advanced level of interfacial nanoarchitectonics achieved by living organisms. Humans have made many examples of rational stacking and functionalization of layered structures by applying the principles of nanoarchitectonics. Yet, the construction of complex functional structures within a single plane still has a lot of mechanisms to understand and challenges to overcome. Therefore, the frontier in forming complex advanced functional structures lies here in lateral nanoarchitectonics.

Here, we review the lateral structural architectures of various functional systems projecting the general principles of lateral nanoarchitectonics into each example. The examples presented are not necessarily selected from well-developed research subjects. We examine the degree of development by referring not only to recent examples of research, but also to examples from several decades ago. In this review, we will discuss the following topics, depending on the size and complexity of the structure: (i) lateral relay activity of molecules and molecular machines on a solid, (ii) Langmuir system: structure formation, (iii) Langmuir system: formational regulation between molecules and bulk, and (iv) Langmuir and liquid interfacial systems: emerging challenges, (v) on an aqueous membrane, and (vi) living cells at the liquid interface. These include extending molecular level functions by on-surface synthesis, monolayers at the air–water interface, formation of supramolecular receptors, 2D molecular patterning, macroscopic manipulation of molecular machines, collective functions of molecular rotors, coupled functions of biomolecular machines, and many more. By considering various interfaces, the current and future development of lateral nanoarchitectonics is assessed. We believe that this review article will provide such an opportunity and help us find new groundbreaking directions in lateral nanoarchitectonics toward advanced functional material organization.

## 2. Lateral relay activity of molecules and molecular machines on a solid

Molecular lateral nanoarchitectonics postulates the most fundamental principles at the level of individual molecules that are crucial for understanding more complex systems. In this section, we summarize the lateral structural extensions and functional linkages and their visualization on the molecular level.

### 2.1. Covalent approach

Atomic and molecular-level nanoarchitectonics on 2D surfaces has been studied in combination with probe microscopy.^44^ For example, an array of diacetylene derivatives adsorbed on a surface can be stimulated with the tip of a probe microscope to form polydiacetylene molecular wires at the desired locations.^45^ There is also an example of using a probe microscope to selectively induce longitudinal oligomerization at a specific location on an array membrane consisting of several molecular layers of C_60_ molecules.^46^ Stimulated and unstimulated locations can be distinguished at the molecular level in 2D space. This technique can create arbitrary molecular-level resolution patterns resulting in the formation of 2D barcodes in a 2D plane. Another example is the use of a chip that can activate surface molecules by removing individual bromine atoms allowing a subsequent surface reaction with fullerene molecules.^47^ In the latter example, an organic reaction can occur at the desired position of the molecule on the surface. This approach is called local probe chemistry.^48^ Thus, lateral nanoarchitectonics with molecular-level resolution can be developed by controlling organic synthesis on surfaces. Examples of such research took place especially in the research field of surface synthesis.^49^ In the following paragraphs, we will discuss some recent examples of this trend.

**Fig. 2:**
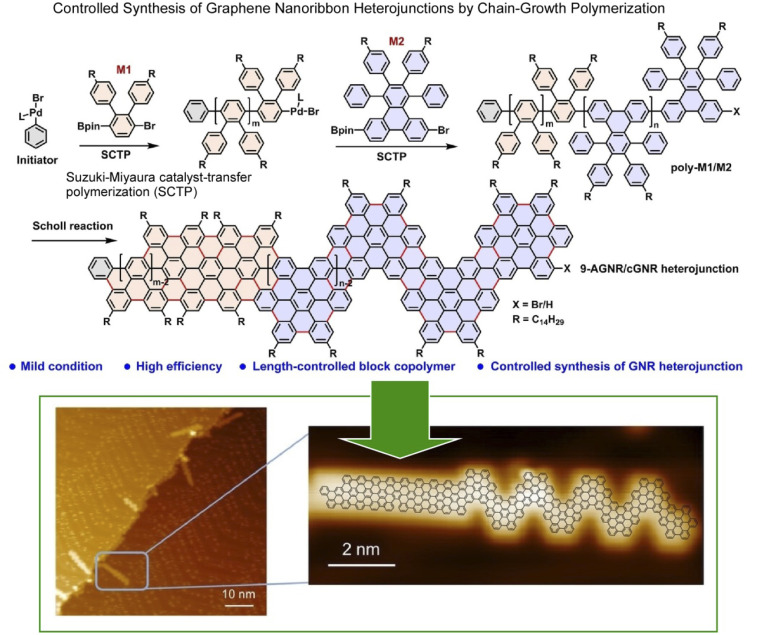
On-surface synthesis of graphene nanoribbon heterojunctions from block polyphenylene precursors by chain-growth polymerization: chemical reaction (top) and molecular-level imaging by *in situ* scanning tunnelling microscopy (bottom). Reproduced under terms of the CC-BY license from ref. 51, 2023 Wiley-VCH.

Graphene nanoribbons are promising candidates for next-generation nanoelectronics.^50^ These structures can be laterally constructed for more advanced nanoarchitectures. In particular, graphene nanoribbon heterojunctions have attracted a great deal of attention because they exhibit exotic topological electronic phases at the heterointerface. Ma, Feng, and co-workers synthesized graphene nanoribbon heterojunctions from block polyphenylene precursors by chain growth polymerization (Fig. 2).^51^ First, precursors of heterojunctions with *N* = 9 armchair graphene nanoribbon segments and chevron graphene nanoribbon segments were synthesized. In the next step, cyclodehydrogenation of the block polyphenylene precursor resulted in the formation of graphene nanoribbon heterojunctions. The process was analysed *in situ* by scanning tunneling microscopy (STM) at the molecular level. The graphene nanoribbon heterojunctions, which combine units with different topologies, enable novel electronic band structure engineering. It can provide new topological electronic states at the interface. Exotic topological states are useful for quantum information processing devices and spintronics. The strategy of projecting lateral nanoarchitectonics into the fabrication of novel graphene nanoribbon heterostructures will provide molecularly designable carbon materials for applications in advanced nanoelectronics devices.

**Fig. 3:**
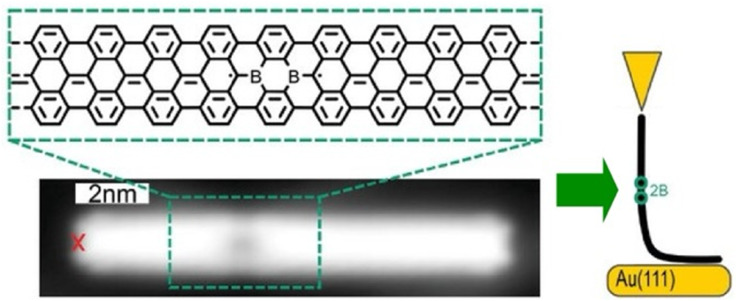
Measurement of quantum transport through borylated graphene nanoribbons suspended between the STM tip and the surface: bottom image, structure of a borylated ribbon segment and STM topography image where the red cross indicates the position from where the ribbon was lifted. Reproduced under terms of the CC-BY license from ref. 52, 2024 Wiley-VCH.

Single-molecule current rectifiers are fundamental building blocks of organic electronics. Friedrich, Pascual, and co-workers reported a study of tuneable current rectification through a designed graphene nanoribbon.^52^ The extraordinary current rectification efficiency was achieved by doping seven-unit graphene nanoribbons with one unit of diboron on a gold substrate with atomic precision. Quantum transport through suspended boronated graphene nanoribbons between the STM tip and the surface was investigated (Fig. 3). Monopolar resonant transport *via* a boron-induced in-gap state embedded in graphene nanoribbons was examined. The presence of the boron moiety confined the valence band, while a quantum-well state was formed in the ribbon. Both the ground and excited states of the quantized bands supported the resonant transport of electrons, which enabled efficient *in situ* tuneable current rectification. The asymmetric position of the quantum dots within the ribbon was comparable to that of the current rectification of asymmetric two-level molecules. This example of molecular nanoarchitectonics represents an innovative approach to precisely manipulate the functionality of molecular electronic states. It opens new avenues for advanced applications in organic electronics.

### 2.2. Supramolecular approach

Functional propagation between molecules arranged in a supramolecular non-covalent arrangement in a 2D plane has also been investigated. For example, Heinrich, Lutz, and co-workers demonstrated molecular cascades by organizing carbon monoxide molecules on a copper(111) surface with atomic precision using low-temperature STM.^53^ In their model, the motion of one molecule triggered the motion of another, resulting in a domino-like cascade motion at temperatures below 6 K. Here, the hopping motion of the carbon monoxide molecules occurred as a result of the quantum tunneling of the molecules between adjacent sites on the surface. The tunneling rate was controlled by adjusting the direction of hopping and interactions with neighbouring molecules. Devices such as logic gates were created by placing molecules at the intersections of the cascade to design multiple AND and OR gates. Overall, the ability to control the direction and speed of molecular motion demonstrated the potential for extremely small logic circuits.

**Fig. 4:**
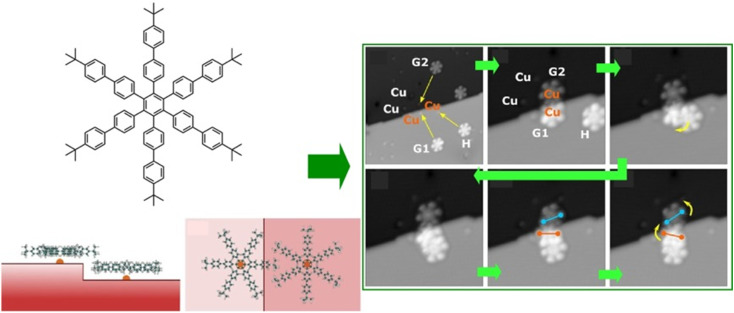
Interlocking structure of three hexa-*tert*-butylbiphenylbenzene molecular gears on a superconducting Pb(111) surface at a different monatomic step: (left) chemical structures of integrated gears and (right) STM images of train motions of the interlocked gears. Reprinted with permission from ref. 54 Copyright 2020 American Chemical Society.

Interlocking molecular machines laterally in a 2D plane has also been demonstrated. This is the creation of molecular gears that move in tandem by bringing multiple molecular rotors into lateral contact in a 2D plane. Gears are universal mechanical elements used in many fields; not only are they key components in basic mechanisms of technology, such as clocks and motors, but they are also essential components of machines that operate in harsh environments, such as nuclear power plants and outer space. General requests are to minimize the energy required for such a machine to function and to reduce its weight for portability. To achieve this, it is imperative that the gears be as small as possible. To this end, molecular gears were fabricated using single-molecule manipulation by STM. The transmission of rotation along the gear train is an essential prerequisite for the construction of molecular mechanical machines. Soe *et al.* reported the interlocking structure of two and three molecular hexa-*tert*-butylbiphenylbenzene gears on a superconducting Pb(111) surface (Fig. 4).^54^ The gears were designed and synthesized with long biphenyl teeth. To minimize the mechanical entanglement between the gears that hinders the transmission of the rotation along the gear train, the advantage of the native monatomic steps on the Pb(111) surface was used. When each molecular gear was placed in the interlocking train at a different monatomic step height on the support surface, a functioning long molecular gear train was constructed. Other measures include designing long, rigid molecular teeth. Alternatively, the interaction between the rotating elements and the surface can be reduced by elevating the gears on the anchor unit. It can be concluded that a sophisticated molecular design is crucial for the interaction of molecular rotors.

**Fig. 5:**
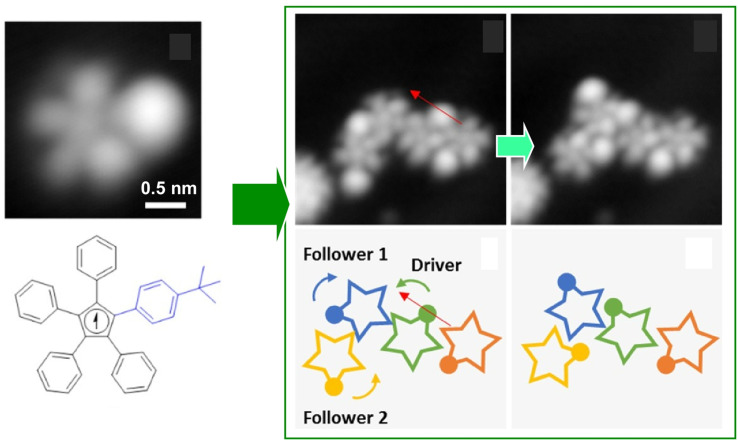
(Left) a molecular gear with a *tert*-butyl group on the tooth; (right) the rotation transmission of three interlocking units from the driver gear to two followers triggered by the tip of the STM at the driver gear. Reprinted with permission from ref. 55 Copyright 2020 American Chemical Society.

It is advantageous for the molecular gear to be equipped with a specific group that facilitates the tip–molecule interaction to enhance the manipulation by STM. Such a marker also enhances the evaluation of rotational behaviour. Therefore, Moresco and co-workers attached a *tert*-butyl group on the end of the tooth of one of the gears (Fig. 5).^55^ With this design, reproducible stepwise rotation of a single gear was achieved. It was also ensured that the rotation of up to three interlocking units could be transmitted: operating the *tert*-butyl tooth of the third gear as a driver at the tip of the STM; an incidental behaviour was created in the transmission of rotation between the three meshing gears. A sequence of rotational transmission along a gear train connecting three molecules was as follows: when a molecule rotated counterclockwise from the driver, a follower molecule rotated 75° clockwise. Simultaneously, another follower molecule rotated 78° counterclockwise. A next-level challenge is the development of a molecular mechanical calculator; the nanoarchitectonics and controllability of rotating a large number of gears in the interlocking train will be crucial for this purpose.

In this section, we have shown several examples of lateral nanoarchitectonics at the molecular level. Through the extensive connection of the desired molecular units to the on-surface synthesis, highly organized structures can be created, allowing the individual molecules to act as electronic devices. Alternatively, a mechanism in which molecular gears move in tandem holds great potential for creating ultrasmall machinery. Further development of these approaches will give rise to tiny mechanical parts with a degree of modification at the molecular level, which is currently at the very basic stage of research. It is clear that these examples represent novel and challenging research fields. The lateral architecture of molecules and their assemblies will lead to the development of a wide variety of molecular technologies in this direction. Many of these examples require both nanotechnology techniques, such as STM observation and molecular metrology, and knowledge of traditional organic synthesis. This is a typical style of nanoarchitectonics approach, *i.e.*, the fusion of nanotechnology and existing materials science.

## 3. Langmuir system: structure formation

The examples discussed in the last section illustrate how lateral nanoarchitectonics and precise molecular positioning, achieved through techniques such as STM, can lead to the creation of highly organized molecular systems and functional devices on solid surfaces. However, molecular positioning and organization are not limited to solid surfaces. In fact, Langmuir systems provide another powerful approach for constructing ordered molecular layers at liquid interfaces. Similar to the control achieved through STM on solid substrates, Langmuir systems allow for the deposition of well-defined molecular layers with nanoscale precision. Moreover, more dynamic lateral functional links occur in membrane structures, such as lipid bilayers in aqueous solution or Langmuir monolayers on aqueous surfaces.^56^ In this section, we will give some characteristic examples of lateral nanoarchitectonics in Langmuir systems, focusing on the fabrication of specific structures.

### 3.1. Biomimic lipid raft model

A functional lateral structure with a particular arrangement and clustering of molecules within the lipid monolayer in a biomembrane is called a lipid raft.^57^ It can be thought of as lateral nanoarchitectonics in lipid membranes that takes place naturally. Lipid rafts are liquid-ordered phases that are islands of ordered lipids that coexist within a liquid-disordered phase. Lipid rafts are responsible for many functions of the membrane. Their precise nature and molecular structure are being investigated with great interest. Using the lipid raft model, it became clear that the specific molecular arrangements of the phospholipid aggregates in the membrane are deeply involved in physiological functions. For example, the relationship between lipid rafts and the function of proteins incorporated into them is of interest. To study most membrane proteins, their insertion into lipid membranes is necessary to fully understand their properties and activity. The successful reconstitution of a protein in a lipid raft model membrane depends on the lateral nanoarchitectonics of the membrane.

As an example of a lipid raft model, Bilewicz and co-workers examined the incorporation of a protein 3-hydroxy-3-methylglutaryl coenzyme A reductase (HMG-CoA reductase) into the mimetic membrane of the lipid raft.^58^ This enzyme is a transmembrane glycoprotein located on the membrane of the endoplasmic reticulum and is responsible for cholesterol biosynthesis in hepatocytes. Model lipid membranes composed of 1,2-dioleoyl-*sn-glycero*-3-phosphocholine (DOPC), cholesterol (Chol), and sphingomyelin (SM) in a 1 : 1 : 1 molar ratio were designed to reproduce lipid systems with compositions and surface properties that mimicked lipid rafts. The Langmuir monolayer, formed by spreading liposomes and proteoliposomes at the air–water interface, was used as a thin lipid raft membrane model to measure reductase activity and monitor statin inhibition. The Brewster angle microscopy images recorded after spreading the liposomes indicate the coexistence of the phases as well as phase separation. It was confirmed that the activity of the reductase membrane was maintained over time in the raft model membrane at the air–water interface. Furthermore, the inhibition process was monitored by changing the concentration of the components of the catalytic reaction. Such studies of membrane proteins in lipid raft model environments are well suited for investigating the underlying mechanisms of lipid–protein interactions. It also provides a model system to understand the effects of other molecules on protein activity.

**Fig. 6:**
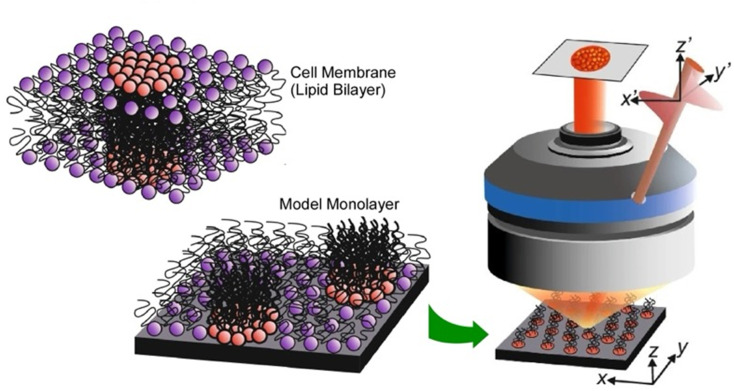
Phase-resolved sum-frequency generation microscopy to analyze micron-scale condensed domains of phospholipid monolayers of mixed chirality as a lipid bilayer raft in 3D space. Reproduced under terms of the CC-BY license from ref. 59, 2024 Springer-Nature.

Analyses of the in-plane structure of lipid raft models also provide an assessment of the in-plane structures that spontaneously form in lateral nanoarchitectonics. Studies of condensed islands in lipid rafts have shown a wide range of sizes and morphologies, suggesting substantial in-plane molecular anisotropy and mesoscopic structural chirality. Thämer and co-workers used phase-resolved sum-frequency generation microscopy to analyse micrometre-scale condensed domains of mixed-chirality model phospholipid monolayers in 3D space (Fig. 6).^59^ In the reported approach, the C–H stretching vibrations of the phospholipid molecules were directly probed. To investigate molecular packing and interactions, monolayers of (*R*)/(*S*)-dipalmitoylphosphatidylcholine and fully deuterated (unsaturated) 1-palmitoyl-2-oleoyl-*glycero*-3-phosphocholine mixed in a 4 : 1 ratio were fabricated. Hyperspectral images of different dipalmitoylphosphatidylcholine-rich domains of the membrane were observed. This imaging technique combined the spectroscopic selectivity to distinguish molecular species with the ability to detect the absolute molecular orientation encoded in the signal phase. The domains showed a curved molecular orientation with helical mesoscopic packing. Both the molecular orientation and helical rotation direction depended on the chirality of the lipid. Different enantiomeric mixtures formed structures that deviated from the mirror symmetry. These facts indicated strong enantioselectivity in the domain growth process. In other words, a fundamental thermodynamic difference between homochiral and heterochiral membranes was indicated. This advance in microscopic vibrational imaging offers promising prospects for further studies of lipid rafts.

### 3.2. Associated receptor and 2D molecular pattern

Lipid rafts, which are formed by the lateral aggregation of lipids in the membrane plane, are interesting biochemical targets. Not only natural lipids, but artificially synthesized amphiphilic molecules have also been used in lateral nanoarchitectonics to generate functional structures by specifically assembling them in a 2D plane. For example, complex molecular sites can be created by lateral nanoarchitectonics on aqueous interfaces. Receptor proteins and enzymes have molecular recognition cavities in which peptide residues are precisely organized. However, artificially reconstructing such active structures is often a very challenging task. Amphiphilic molecules with relatively simple peptide residues and amphiphilic molecules with various functional groups can be assembled in the Langmuir membrane plane to create sophisticated receptor structures in nanoarchitectonics.^60^

**Fig. 7:**
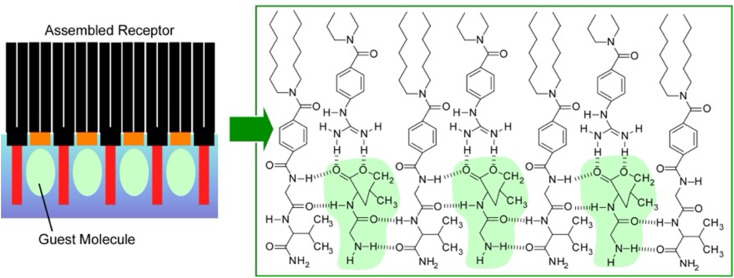
Associated receptor at the air–water interface: an equimolar mixed monolayer of a dioctadecylglycylglycinamide amphiphile and a functional amphiphile with guanidinium groups for the optimal recognition structure of a water-soluble dipeptide (left: guest molecules and right: green labels).

Fig. 7 shows an example of an equimolar monolayer consisting of a dioctadecylglycylglycinamide amphiphile and an amphiphile functionalized with guanidinium groups.^61^ In this mixed monolayer, the optimal recognition structure was spontaneously formed by molecular assembly in the presence of a water-soluble dipeptide (Gly–Leu). Langmuir isotherm analysis of the binding behaviour of Gly–Leu to this mixed monolayer showed that the binding site of a single Gly–Leu molecule was formed cooperatively by the two monolayer components. The binding constant was 6400 M^−1^ which was higher than the binding constant for the monolayer of the glycylglycinamide amphiphile alone. This means that simple functional groups assembled on a supramolecular basis can show great capability through their cooperative action. When benzoate amphiphiles were used as mixed components instead of functional amphiphiles with guanidinium groups, the binding constants decreased. Obviously, the guest binding efficiency was regulated by the component molecules being nanoarchitectonized. It is important to create a suitable configuration state of the host functional group for strong hydrogen bonding with the guest. Such a lateral nanoarchitectonics approach has the great advantage of being able to accommodate the recognition of diverse guests by changing the functional groups of the components and their combinations. In particular, the high binding efficiency demonstrated here could lead to the sensitive detection of a wide variety of aqueous peptides. This transformation should be useful for sensing physiologically important peptides, such as peptide hormones and neuropeptides. Since hydrogen bonding plays an essential role, the system could be applied to other types of biologically important guests. For example, nucleotide mimics can create recognition domain peptide structures on the surface of monolayers for enzyme cofactors. Model systems could be designed for the recognition of sugars by lectins or gangliosides by hemagglutinin.

**Fig. 8:**
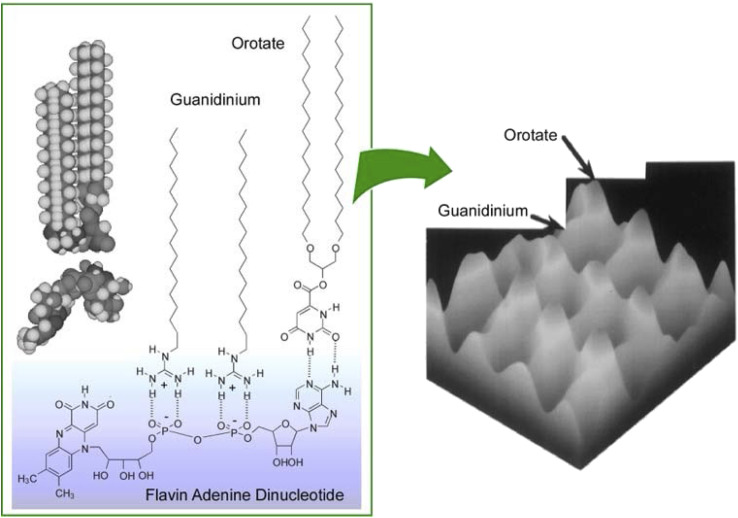
2D molecular pattern with a specific molecular arrangement in the monolayer surface: (left) multisite molecular recognition of guanidinium and orotate amphiphiles with water-soluble flavin adenine dinucleotide; (right) an AFM image of the mixed monolayer transferred from a flavin adenine dinucleotide solution with a periodic pattern consisting of two methyl peaks of different heights.

It was experimentally^62^ and theoretically^63^ proven that molecular recognition by molecular interactions such as hydrogen bonding is stronger at the air–water interface than in the bulk aqueous phase. In addition to the recognition of peptides already discussed, molecular recognition at the air–water interface has been reported for sugars,^64^ amino acids,^65^ nucleobases,^66^ and nucleotides.^67^ When water-soluble guest molecules with multiple different moieties or functional groups are used, the corresponding amphiphiles for recognition may be bound separately at each location. In other words, a single water-soluble guest molecule can specifically interact with different types of membrane components. This ability allowed the creation of a 2D molecular pattern with a specific molecular arrangement on the surface of the monolayer.^68^ As shown in Fig. 8, water-soluble flavin adenine dinucleotides had multiple complementary hydrogen bond recognition sites. As a result, multisite molecular recognition occurred at the air–water interface. In this case, guanidinium and orotate amphiphiles were used as recognition host molecules. Mixed monolayers of these amphiphilic molecules were transferred onto mica and their surfaces were observed by atomic force microscopy (AFM). The monolayers transferred from pure water showed a surface of uniform height and a periodic hexagonal pattern consisting of only one type of methyl peak. On the other hand, the AFM image of the mixed monolayer transferred from a flavin adenine dinucleotide solution showed a periodic pattern consisting of two methyl peaks of different heights. The 2D molecular patterning of the latter resulted from the rearrangement of the monolayer components based on specific recognition by the flavin adenine dinucleotide template molecule. Through binding of the flavin adenine dinucleotide to the mixed monolayer, the recognition functional groups of the two amphiphilic molecules were placed at the same height. This resulted in a height difference between the terminal methyl groups of the two amphiphilic molecules. The 2D arrangement of the interacting groups in the template molecule was translated into a height pattern of alkyl chains. This observation is proof that lateral nanoarchitectonics can effectively target an appropriate molecular design of templates to organize regularly repeating patterns in various monolayers.

### 3.3. Supramolecular polymer and gel fiber

**Fig. 9:**
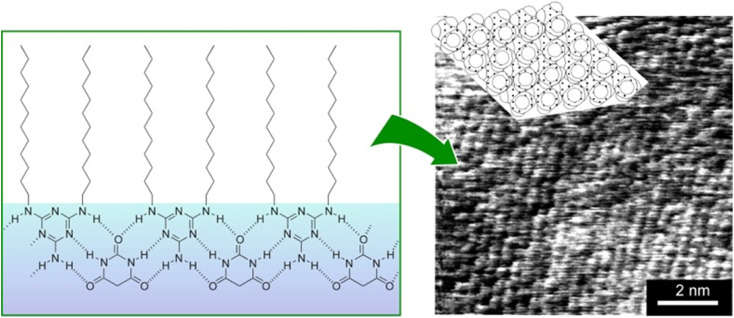
Supramolecular polymer at the air–water interface: (left) chemical structures of the supramolecular polymer of dialkylmelamine (2-amino-4,6-di(dodecylamino)-1,3,5-triazine) molecules with aqueous barbituric acid; (right) an AFM image of the transferred supramolecular polymers on a mica surface showing that the terminal methyl groups are regularly arranged.

Supramolecular polymers are a class of polymer-like materials in which individual building units assemble non-covalently *via* intermolecular forces such as hydrogen bonding, π–π interactions, and others.^69^ Koyano *et al.* developed a supramolecular polymer at the air–water interface by deploying a monolayer of long-chain dialkylmelamine in an aqueous barbituric acid solution (Fig. 9).^70^ The surface pressure–molecular area (π–*A*) isotherms indicated that long-chain dialkylmelamine (2-amino-4,6-di(dodecylamino)-1,3,5-triazine) molecules created well-packed monolayers at the air–water interface. When the aqueous phase contained barbituric acid, the molecular area increased and barbituric acid molecules were inserted to form a supramolecular polymer. Supramolecular polymer formation was monitored with infrared (IR) spectroscopy by focusing on the signals of hydrogen bonds between long-chain dialkylmelamines and barbiturates. The binding constant of barbituric acid to long-chain dialkylmelamine monolayers was large enough at 3000 M^−1^, providing the necessary stiffness of the resulting supramolecular polymers. The structure of the monolayer transferred to mica was observed by AFM. The monolayers transferred from the pure monolayer were fragile and unsuitable for observation. However, long-chain dialkylmelamine monolayers transferred from barbituric acid solution onto mica plates were observed by AFM, showing a regular arrangement of the terminal methyl groups. This method of forming supramolecular polymers by the association of two components at the air–water interface can be utilized with a variety of combinations of components, and it therefore represents a powerful tool for lateral nanoarchitectonics.

Marchi-Artzner *et al.* investigated the behaviour of hydrogen-bonded supramolecular polymers of 2,4,6-triaminopyrimidine and barbituric acid linked to tetraoxyethylene spacers at the air–water interface.^71^ In this case, the resulting supramolecular polymer possessed a flexible structural design because of the presence of tetraoxyethylene spacers between the hydrogen-bonding functional group and the alkyl chain. The behaviour of the supramolecular polymer at the air–water interface was investigated using fluorescence microscopy in addition to π–*A* isotherms, AFM, FT-IR, and XPS. The effects of pH and ionic strength of the aqueous phase were also investigated. In particular, the latter results indicated an electrostatic contribution from the acid–base properties of 2,4,6-triaminopyrimidine and barbituric acid in addition to hydrogen bonding interactions. The results showed that the formation of supramolecular polymers was highly efficient in the presence of tetraoxyethylene spacers. Moreover, it can be generalized that the use of spacers provides the resulting structures with flexibility and freedom in their design and functionalization.

**Fig. 10:**
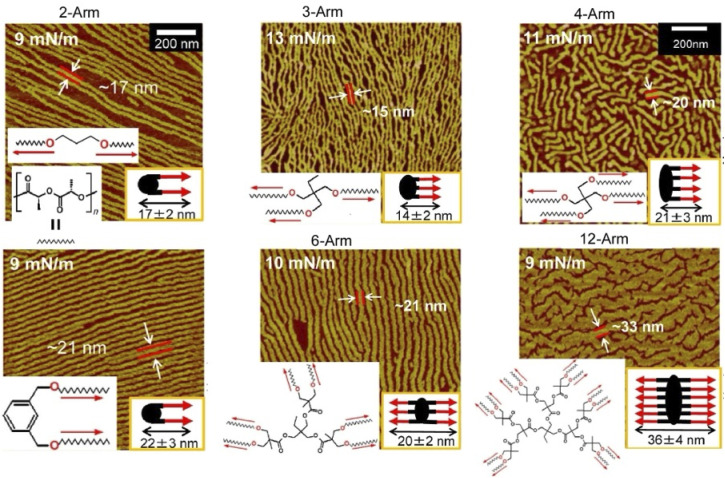
AFM images of extended chain crystals of the synthesized star-shaped poly(l-lactide) with 2–12 arms in Langmuir monolayers where two poly(l-lactide) chains then crystallize into folded lamellae with a central diol unit aligned in the same direction. Reprinted with permission from ref. 72 Copyright 2023 American Chemical Society.

Rather than specifying a rigid structure at the interaction site, as in supramolecular polymers, lateral nanoarchitectonics can be advanced at the air–water interface through more ambiguous and flexible molecular associations. Kumaki and co-workers synthesized star-shaped poly(l-lactide) with 2–12 arms by polymerizing l-lactide with various polyols as initiators to induce crystalline behaviour in monolayers (Fig. 10).^72^ As supported by AFM analysis, poly(l-lactide) crystallized by forming extended chain crystals in Langmuir monolayers. The advantage of analysing the monolayers was in the fact that the chain packing was characterized by determining the thickness of the lamellae. On spreading double-stranded poly(l-lactide) on the surface of water in a diluted state, isolated chains floated at the air–water interface. Upon compression, the two poly(l-lactide) chains first formed a condensed amorphous monolayer. Subsequently, the two poly(l-lactide) chains crystallized into folded lamellae with central diol units aligned in the same direction. Crystallization proceeded *via* a condensed amorphous state. Poly(l-lactide) consisting of two to four arms crystallized with all arms aligned in the same direction and folded with a central polyol unit. On the other hand, poly(l-lactide) consisting of 6 and 12 arms crystallized with half of both arms extending in opposite directions from the centre. This was probably due to steric obstacles in the crowded arms. In both systems, the poly(l-lactide) arms showed a strong tendency to crystallize in the same direction. Such observations and the elucidation of the general trend are important for lateral nanoarchitectonics at the air–water interface using a variety of polymers.

**Fig. 11:**
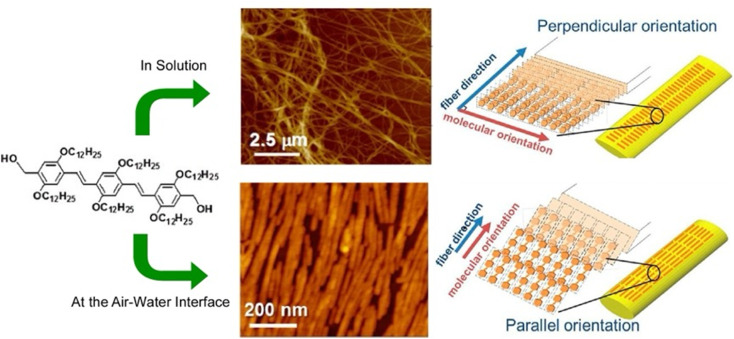
Controlled 1D assemblies with different internal molecular orientations: (top) entangled fibers processed in solution; (bottom) aligned nanorods assembled at the air–water interface. Reprinted with permission from ref. 74 Copyright 2014 American Chemical Society.

Gels are formed when small molecules aggregate to form long-range structures. In gels, self-assembled one-dimensional (1D) fibers incorporate solvent and form 3D structures.^73^ Specifically, a linear π-gelator self-assembles into an entangled fiber with molecules aligned perpendicular to the long axis of the fiber. However, with this general approach, it is difficult to orient the gelator molecules parallel to the long axis of the 1D structure. Lateral nanoarchitectonics at the air–water interface can offer a suitable solution. Sakakibara *et al.* aligned nanorods composed of oligo(*p*-phenylenevinylene)-derived π-gelators at the air–water interface and studied the molecular orientation within the aligned rods (Fig. 11).^74^ The nanorods were 340 ± 120 nm in length and 34 ± 5 nm in width and showed the orientation of oligo(*p*-phenylenevinylene) molecules parallel to the long axis of the rods. Upon increasing surface pressure, the rods aligned in one direction to fill the voids in the monolayer effectively. Near-field scanning optical microscopy revealed that local photoexcitation led to different excited-state properties. Polarized fluorescence spectra of the aligned rods showed marked anisotropy. The polarization intensity ratio (parallel/perpendicular) was 2.4. Interestingly, this was completely opposite to the fluorescence polarization measurements of the alignment of entangled fibers of oligo(*p*-phenylenevinylene)-type gels made in solution, which showed a strong fluorescence intensity in the vertical direction. Long-range excitation energy transfer occurred in the entangled fibers of solution gels, causing fluorescence quenching. In contrast, fluorescence was greatly enhanced in nanorods aligned at the air–water interface. The overall results demonstrated that entangled gel fibers with vertically aligned molecules are suitable for excitation energy transfer, while nanorods with a parallel alignment of molecules are suitable for charge transport. In chromophore supramolecular assemblies, the packing mode of molecules at the nanoscale is an important factor that controls the photophysical and energy transport processes of excited states. Understanding the mechanism of excitation energy transfer in 1D molecular assemblies may help design supramolecular structures with improved charge transport properties. Lateral nanoarchitectonics at the air–water interface can yield efficient functional structures.

In this section, we introduced several examples of lateral nanoarchitectonics at the air–water interface, focusing primarily on structure creation. The freedom of motion at the air–water interface and the limited extent of spreading allowed for specific 2D nanoarchitectonics. Its effective importance is exemplified by the biochemical role of lipid rafts. Molecular recognition at the interface can create patterns with molecular-level structural precision. Long-range specific structures can be built by crystallization of polymers and alignment of gel fibers. Although perhaps inferior to molecular nanoarchitectonics on solid surfaces in terms of structural accuracy and specificity, the air–water interface is a very promising venue for lateral nanoarchitectonics with long-range extensions.

## 4. Langmuir system: formational regulation between molecules and bulk

The air–water interface is a very specific functional environment, given its dimensionality and asymmetry of motion. In the lateral direction, the air–water interface extends macroscopically, and its motion is at the visual level. On the other hand, in the thickness direction, it is closed at the molecular level. The liquid interface, including the air–water interface, bridges the molecular level and macroscopic phenomena in terms of both structures and functions.^75^ This section will focus on macroscopic and molecular functional regulation.

### 4.1. Functional regulation from macro to molecule

**Fig. 12:**
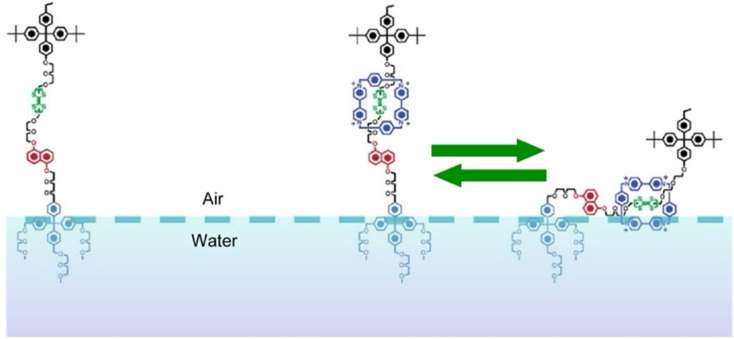
The molecular shuttle with the stationary recognition sites of a tetrathiafulvalene unit and a 1,5-dioxynaphthalene ring unit and a tetracationic cyclophane, cyclobis(paraquat-*p*-phenylene), as the shuttle at the air–water interface. Reprinted with permission from ref. 77 Copyright 2004 American Chemical Society.

The molecular machines operating at the air–water interface attract a great deal of attention.^76^ Frank, Stoddart, and co-workers built a so-called molecular shuttle at the air–water interface.^77^ In their work, the molecular shuttle was derived from a rotaxane structure, as shown in Fig. 12. The stationary recognition sites consisted of units of tetrathiafulvalene and 1,5-dioxynaphthalene rings. A tetracationic cyclophane, cyclobis(paraquat-*p*-phenylene), served as the shuttle. The amphiphilic structure suitable for operating at the air–water interface was given by the choice of stopper. The end close to the tetrathiafulvalene unit was a hydrophobic tetraarylmethane stopper, while the end close to the 1,5-dioxynaphthalene ring unit was terminated by a hydrophilic tetraarylmethane stopper, representing an amphiphilic bistable [2]rotaxane-type molecular shuttle. Using Langmuir film balance, the effects of varying compression rate and subphase temperature were investigated. Analysis of Langmuir films and LB films transferred onto Si substrates suggested that the rotaxane existed in a skeletal conformation at an acute angle to the air surface or to the surface. The pronounced hydrophilicity allowed the cyclophane to be tightly bound to the subphase, resulting in a folded or tilted conformation. The creation of such molecular superstructures will have important implications for molecular electronic devices based on bistable amphiphilic [2]rotaxanes.

As an environment for driving molecular machines, the air–water interface provides a unique medium. Molecular machines are typically driven by irradiation of light, heat energy, chemical reactions, and redox reactions. These external sources stimulate the molecules which induce the propulsion of molecular machines. On the other hand, achieving the propulsion of molecular machines by applying mechanical stimuli is challenging. The only way to directly touch a molecule is to bring it into contact with a tip of a probe microscope. If macroscopic mechanical stimuli, such as hand motions, are to be transmitted to molecules, controlled molecular machines can be operated by hand-motion-like actions. When macroscopic mechanical stimuli such as compression and expansion are applied to monolayers on the air–water interface, the molecules in the monolayer can deform mechanically in response. In lateral nanoarchitectonics at the air–water interface, molecular machines are arranged as monolayers and macroscopic mechanical stimuli can be used to manipulate the molecular machines within the monolayer.^78^ Since mechanical manipulation is induced macroscopically, typically by hand movement-like stimuli, this technology is referred to as hand-operating nanotechnology.^79^

**Fig. 13:**
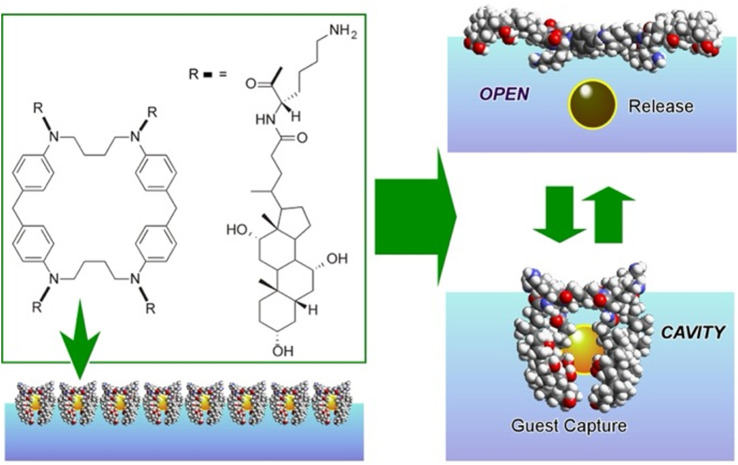
A structure of steroid cyclophane consisting of a cyclic core of 1,6,20,25-tetraaza[6.1.6.1]paracyclophane with four cholic acids linked in its monolayer to exhibit capability of reversible guest molecule capture by external mechanical forces.

A representative example is shown in Fig. 13.^80^ The presented molecular machine was derived from steroid cyclophane; in particular, it consisted of a cyclic core of 1,6,20,25-tetraaza[6.1.6.1]paracyclophane with four cholic acids linked by a flexible l-lysine spacer. When the steroid cyclophane was deployed as a monolayer at the air–water interface, it took on a spreading conformation with the hydrophilic part of the cholic acid on the water surface. When this monolayer was mechanically compressed, the arms bent and took on a cavity-type conformation. During this change, guest molecules in the aqueous phase were trapped. Capture and release of the guest were reversibly repeated and controlled by manipulating the molecular machine by macroscopic mechanical mutation. Langmuir monolayers at the air–water interface represent excellent evaluation media because they can be easily compressed and expanded while monitoring molecular area and surface pressure.

**Fig. 14:**
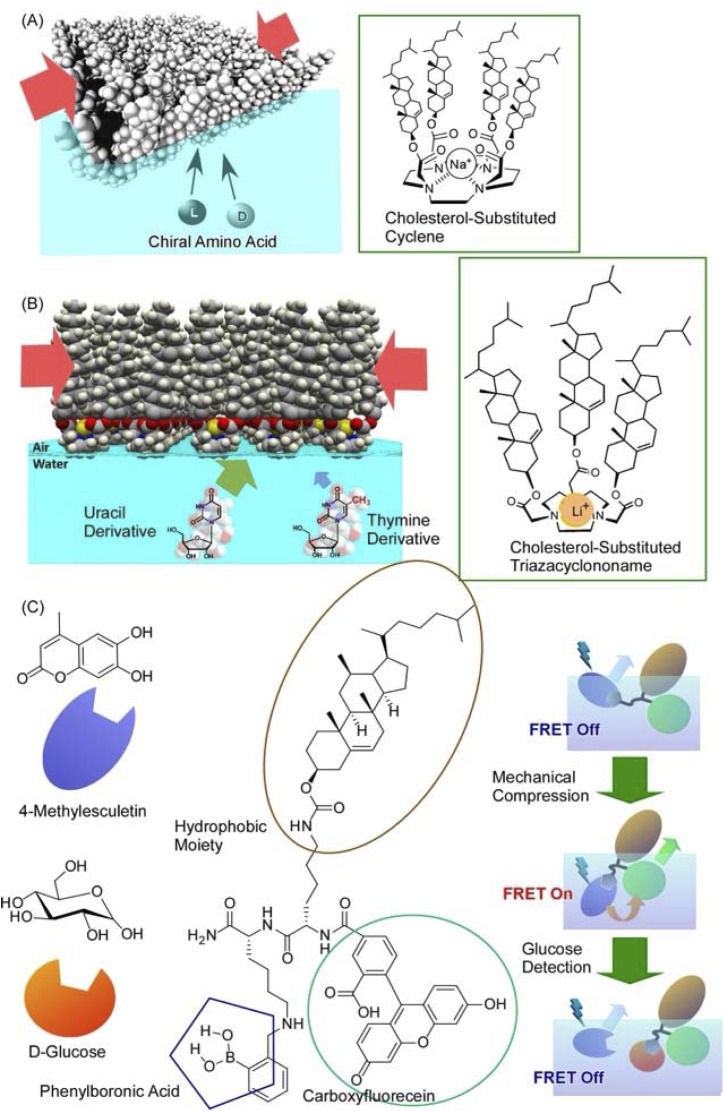
Mechanical tuning of various molecular receptors at the air–water interface: (A) polycholesteryl-substituted cyclen complexes for chiral recognition of aqueous amino acids; (B) cholesterol-armed triazacyclononan for discrimination between thymine and uracil derivatives; (C) receptor for indicator displacement assay of glucose binding with controls of Förster resonance energy transfer (FRET). Reprinted with permission from ref. 81 Copyright 2006 American Chemical Society, ref. 82 Copyright 2010 American Chemical Society, and ref. 83 Copyright 2012 Wiley-VCH.

Not only such dynamic conformational changes, but subtle conformational tuning of receptor molecules is also possible at the air–water interface. Fig. 14A shows polycholesteryl substituted cyclen complexes organized into monolayers as receptor molecules to investigate the binding of amino acids in the aqueous phase.^81^ The receptor monolayer was an aggregate of chiral molecules and the binding of chiral amino acids resulted in diastereomer formation. The enantioselectivity was successfully reversed in the molecular recognition of amino acids by macroscopic lateral pressure. When the monolayer was compressed, the binding constants of the amino acids increased, and, in the case of valine, a reversal of chiral selectivity from the d- to the l- form was observed. The selectivity of the monolayers varied with slight differences, depending on the structure of the amino acid: a feature comparable to the delicate function of enzymes and receptors in living organisms. A similar methodology was applied to one of the most challenging biomolecular recognition problems, namely the discrimination between thymine and uracil (Fig. 14B).^82^ The design and synthesis of enzyme-like artificial hosts for this purpose is extremely difficult. Using a Langmuir monolayer at the air–water interface, a simple receptor molecule was mechanically adapted to determine the optimal point of molecular recognition. Cholesterol-armed triazacyclononan was used as the receptor molecule, and its Langmuir monolayer was compressed in the absence and presence of Li^+^ cations for structural tuning. Under optimized conditions, uracil recognition was achieved approximately 64-fold more selectively than that of thymine. Furthermore, the indicator displacement assay, one of the sensing strategies, was applied to the Langmuir monolayer system (Fig. 14C).^83^ This approach took advantage of the phenomenon of competitive binding of the indicator and guest to complementary sites on the receptor molecule. Mechanical compression was applied to the receptor monolayer at the interface to facilitate the indicator displacement assay. The Förster resonance energy transfer (FRET) between the receptor and the indicator was switched on by this compression; the addition of d-glucose displaced the indicator and effectively quenched the FRET process. The guest concentration was inferred by measuring the ratio of fluorescence intensities *in situ*. Such a link between functional groups in a monolayer is undoubtedly an important clue for the future development of lateral nanoarchitectonics.

In many instances, the molecular recognition mode was considered uniquely with reference to a stable structure such as the crystal structure. However, the addition of the element of dynamically searching for the optimal structure can bring out the potential of the receptor molecule. Molecular recognition began with the primary recognition of guests by host molecules such as crown ethers and cyclodextrins.^84^ This is the basis of supramolecular chemistry. A mode of switching selectivity by isomerization of the host was incorporated later.^85^ In fact, most current molecular machines are based on this switching of stable states.^86^ The interface-based method presented here tunes between a myriad of states to find the optimal structure.^87^ Organic molecules are known for their flexibility; to think of them uniquely in terms of a crystal-like structure or to consider only a few states is not a legitimate use of their potential. Organic molecules can demonstrate their potential capabilities by continuously changing their structures and tuning their functional properties. For this purpose, it is necessary to tune the molecular structure mechanically. It is envisioned as an effective method to link macroscopic behaviour with molecular functions in an interfacial environment.

### 4.2. Functional regulation from molecule to macro

As demonstrated in the above example, the Langmuir monolayer system can couple macroscopic phenomena with those at the molecular level. *In situ* lateral nanoarchitectonics is important for function expression. The above example converted macroscopic mechanical stimuli into recognition functions at the molecular level. Conversely, it is also possible to accumulate molecular-level stimuli at the liquid interface (not necessarily at the air–water interface), which can induce phenomena at the macroscopic level.

**Fig. 15:**
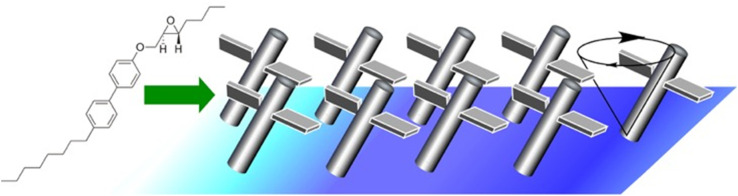
Coherent collective precession of molecular rotors with chiral propellers using liquid crystalline phases at the liquid interface.

Synthetic molecular motors are subject to thermal fluctuations. For this reason, they are likely to be unable to perform useful functions on their own. A mechanism is needed to amplify the motion of a single molecule to a level that is distinguishable from the thermal background. Condensing molecular motors into soft-ordered phases such as interfacial films or liquid crystals is a possible approach to solve it. Tabe and Yokoyama investigated the coherent collective precession of molecular rotors with chiral propellers using liquid crystalline phases at the liquid interface (Fig. 15).^88^ Here, a condensed layer of molecular rotors was developed at the glycerol–air interface. The monolayer consisted of simple rod-like molecules with chiral propellers. As a chiral liquid crystal monolayer, it underwent coherent molecular precession driven by the transmembrane movement of water molecules. As a result, spatiotemporal patterns of molecular orientation were observed. Interestingly, reversing either the molecular chirality or the direction of water molecule movement reversed the direction of rotation associated with the switch from expansion to convergence of the target pattern. Thus, a liquid crystalline phase with only soft-directional order was evaluated as the optimal medium to assist molecular motors in manifesting their individual motion in a collective manner. In principle, the constituent chiral molecules were supposed to rotate in the flow field, even when isolated. However, they tended to be overwhelmed by thermal noise because their molecular masses were not large enough to sufficiently overcome the thermal amplitude. Cooperative motion, as shown here, amplified the fine individual molecular motion to macroscopic scales. This paves the way for lateral nanoarchitectonics in the design of artificial motile molecular systems.

Żywociński, Hołyst, and co-workers reported the collective precession of molecular rotors on liquid surfaces.^89^ The effect of the molecular structure was studied on Langmuir monolayers of four ferroelectric liquid crystals. In two of them, only polar groups were attached to the water surface, with the chiral groups located well above the interface of the elongated molecules. In the other two ferroelectrics, the polar and chiral groups were in close proximity, so that the chiral groups were also attached to the water surface or submerged in water. Comparative experiments showed that the system exhibited collective rotation induced by water evaporation only when the chiral groups of the ferroelectric liquid crystals in the Langmuir monolayer were not attached to the interface and remained in air. In contrast to the linear flow of water, the collective precession behaviour was closely related to the position of the chirality centre relative to the air–water interface. In other words, molecular nanoarchitectonics, which is perpendicular to the interface, can control the collective precession that develops laterally. Cooperative rotation is an example where individual molecular motions are amplified to the mesoscale.

Molecular motion can also be detected as macroscopic wave propagation at the interface. Tabe *et al.* reported photoinduced travelling waves in condensed liquid crystalline Langmuir monolayers composed of azobenzene derivatives.^90^ The presented system was studied using simultaneous microscopy, which enabled the detection of the tilt and azimuthal components of the molecular orientation separately. The dependence of wave generation and propagation on excitation power, symmetry conditions, temperature, and molecular density was determined for azobenzene compounds. Nonequilibrium dynamic patterns were observed in ordinary fluids, semiconductors, and chemical reaction systems in solution and on solid surfaces. In contrast to these classical systems, the reported travelling waves in photoexcitable condensed Langmuir monolayers played an essential role in the pattern formation process because of short-range intermolecular interactions. The rod-shaped molecules in their monolayer were coherently tilted from the layer normal. They were irradiated with weak light to induce *trans*–*cis* photoisomerization. Spatiotemporal periodic oscillations of the molecular azimuthal angles propagated as a 2D azimuthal wave. This wave formation took place at the asymmetric interface, where the film exhibited a broken vertical symmetry. This wave formation occurred only when the chromophore was continuously excited near the long wavelength end of its absorption, and photoisomerization was repeated between the *trans* and *cis* forms. This wave was associated with periodic rotation of the azimuthal angle of the molecule and was not accompanied by tilt vibrations. Langmuir monolayers composed of various azobenzene derivatives exhibited similar travelling waves with velocities proportional to the excitation power. For the wave to propagate over long distances, it had to undergo frequent conformational changes between the *trans* and *cis* states while maintaining an average *cis* ratio of a few percent. As a result, the coupling between the anisotropy of the excitation and the breaking of symmetry with respect to the film plane plays a major role in determining the direction of the wave.

In the above two sections, we explored the possibilities of lateral nanoarchitectonics, focusing on functions in the Langmuir system. A characteristic feature of the series of systems was the coupling of molecular motion with macroscopic functions and properties. At soft, 2D interfaces with degrees of freedom, the macroscopic and the nano-phenomena are coupled. In a sophisticated system such as a monolayer on an aqueous surface, the evaluation of such a system is easier than in a 3D system. At present, the functionality is limited to the level of nano-macro coupling, but the pursuit of lateral functional linkage represents a pioneering challenge. Controlling FRET phenomena by 2D mechanical compression and the propagation of molecular motion as waves can be taken as early examples in this direction.

## 5. Langmuir and liquid interfacial systems: emerging challenges

Langmuir and liquid interfacial systems also provide various opportunities for emerging sciences. For example, attempts have been made to create metal–organic frameworks (MOFs)^91^ and covalent organic frameworks (COFs)^92^ in a liquid interface environment. This can be seen as an example of the application of lateral nanoarchitectonics extending the regular structure horizontally. Another example where lateral nanoarchitectonics can help to elucidate crucial mechanisms is in the field of nano- and microrobotics. Nano- and microrobots are autonomous nano/microscopic objects that possess propulsion abilities and are able to perform given tasks.^93^ Such emerging challenges will be introduced in detail in this section. Some of the examples may not be directly related to nanoarchitectonics. However, such examples are inclusively described here for possibilities in emerging and future challenges.

### 5.1. Interfacial MOF and COF

**Fig. 16:**
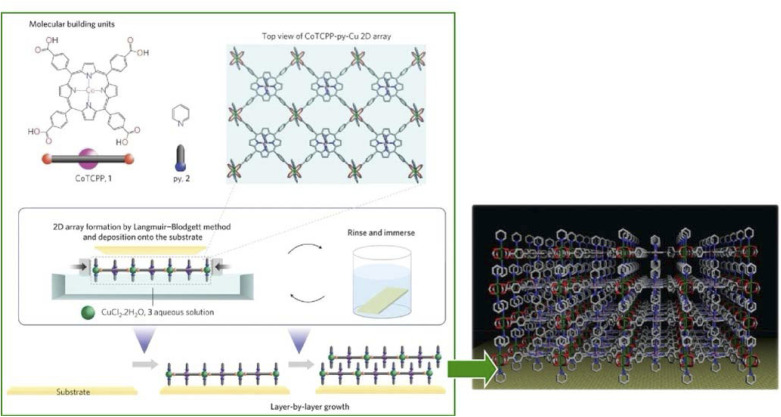
MOF monolayers obtained by developing 5,10,15,20-tetrakis(4-carboxyphenyl)porphyrinato-cobalt(ii) and pyridine on a solution of CuCl_2_·2H_2_O in chloroform/methanol that is multilayered by a sequential LbL stacking procedure. Reprinted with permission from ref. 95 Copyright 2010 Springer-Nature.

The synthesis of ultrathin MOF films and their rational assembly can yield highly ordered microporous materials with a well-controlled growth direction and film thickness. The environment at the liquid interface provides flexibility and high controllability necessary for nanoarchitecting the MOF structures.^94^ Makiura, Kitagawa, and co-workers reported the nanoarchitectonics of fully preferentially oriented MOF nanofilms composed of metalloporphyrins at room temperature (Fig. 16).^95^ In their work, the LB method with complex chemistry was combined to prepare MOF ultrathin films. The combination of various modular processes represented a typical approach of nanoarchitectonics. MOF films consisting of 2D sheet incorporated metal-coordinated pyridine molecules were prepared by developing 5,10,15,20-tetrakis(4-carboxyphenyl)porphyrinato-cobalt(ii) and pyridine in a solution of CuCl_2_·2H_2_O in chloroform/methanol. The principles of lateral nanoarchitectonics enabled the formation of 2D MOF sheets that were subsequently transferred onto a substrate. In the next step, the principles of vertical nanoarchitectonics allowed for a sequential LbL stacking to form a highly organized multilayered film consisting of metalloporphyrin building blocks and metal-ion junctions. This was a nice example of the combination of lateral and vertical nanoarchitectonics that led to the production of MOF nanofilms of arbitrary thickness. It is worth noting that these nanoarchitectonics strategies are versatile, and it would be possible to stack individual layers of different types of MOFs to obtain complex ordered heterostructures. This nanoarchitectonics methodology is suitable for creating heterostructures with smooth interfacial junctions and will be a powerful method for obtaining integrated device systems.

Single molecule magnets are an attractive target for memory device fabrication since they are able to dramatically increase information storage capacity. Regular arrays of single molecule magnets must be constructed on a substrate for facile access to individual single molecule magnets. To meet this requirement, Horii and co-workers fabricated a MOF nanosheet-based single molecule magnet by applying the principles of lateral nanoarchitectonics.^96^ The single molecule magnet consisted of Tb^3+^ sandwiched between 5,10,15,20-tetrapyridylporphyrinato and phthalocyaninato ligands. Phthalocyaninato-porphyrinato-terbium(iii) double-decker single molecule magnets reacted with Pd^2+^ ions at the air-liquid interface upon the formation of regularly and preferentially oriented mechanically robust MOF nanosheets. X-ray magnetic circular dichroism measurements revealed that the MOF sheets exhibited perpendicular magnetic anisotropy. It had significant advantages in the high concentration of single molecule magnets and in the regularity of the structure. In addition, compared to conventional methods in terms of substrate-independent structures, this approach seems to be convenient and highly efficient; it holds great potential for the construction of molecular magnetic memory devices.

**Fig. 17:**
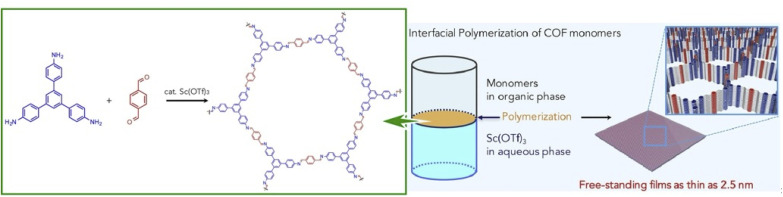
COFs with imine bonding obtained by interfacial polymerization of 1,3,5-tris(4-aminophenyl)benzene and terephthalaldehyde as monomers using Sc(OTf)_3_, a Lewis acid catalyst, resulting in nanoarchitectonics of a continuous COF film at the interface. Reprinted with permission from ref. 97 Copyright 2018 Elsevier.

2D COFs are crystalline polymers with a lattice-like structure that have been applied for the fabrication of energy storage devices and water purification systems. Similar to the preparation of MOF structures, COFs can be efficiently synthesized with the assistance of nanoarchitectonics. Matsumoto *et al.* reported a method for forming COFs at the oil–water and air–water interfaces (Fig. 17).^97^ In particular, imine-bonded COFs were obtained by interfacial polymerization of 1,3,5-tris(4-aminophenyl)benzene and terephthalaldehyde monomers using Sc(OTf)_3_, a Lewis acid catalyst. Sc(OTf)_3_-catalyzed polymerization proceeded rapidly at room temperature with a low catalyst loading. The critical advantage of using the principles of lateral nanoarchitectonics was in the fact that the accumulation of the catalyst and monomer at the interface induced site-selective polymerization, which led to the formation of a continuous COF film. Moreover, there were fewer design constraints on the monomer, such as the need to dissolve the monomer in different phases. Continuous COF films of large area (several cm^2^) were obtained with the thickness ranging from 100 μm to 2.5 nm. The large area, controlled pore size, and tuned molecular composition were evaluated to be promising for nanofiltration applications. The COF membranes were transferred to polyethersulfone supports and tested for water purification from model organic contamination. The interfacial polymerization of imine-bound COFs with Sc(OTf)_3_ shown here is expected to be applicable for the preparation of other functional COF thin films, such as electronically active COFs.

**Fig. 18:**
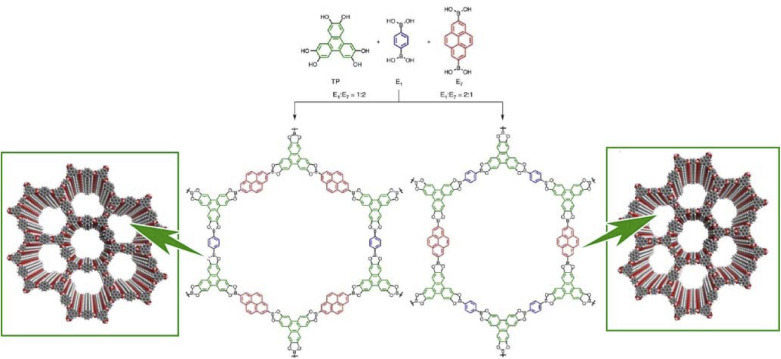
COF synthesis based on multicomponent condensation systems with different component ratios. Reproduced under terms of the CC-BY license from ref. 98, 2016 Springer-Nature.

Lateral nanoarchitectonics at the interface is not the only approach toward the synthesis of COFs. Jiang and co-workers reported the preparation of electronically active multicomponent COFs in a fully liquid environment (Fig. 18).^98^ The synthesis was based on multicomponent [1 + 2] and [1 + 3] condensation systems using one knot and two or three linker units. The resulting hexagonal and square multicomponent COFs comprised asymmetrically tiled organic units that formed anisotropic frameworks and peculiarly shaped pores. Yaghi and co-workers applied the principles of nanoarchitectonics to synthesise imine-bonded COFs by combining hexaaminophenylbenzene, tetragonal tetrakis(4-aminophenyl)ethane, and trigonal 1,3,5-tris(*p*-formylphenyl)benzene.^99^ As a result, 2D COFs with unprecedented topology were obtained. The incorporation of three different types of linkers of different connectivities within the 2D COFs enabled precise control of the resulting geometry; the multicomponent COF had three types of vertices and two types of edges. This high degree of complexity can extend the range of 2D COF structures. By developing such a multicomponent COF structure with the assistance of lateral nanoarchitectonics at the interface, it will be possible to create functional systems that develop rational functional coordination within a 2D plane. It is expected that the vectorial transfer of electrons and the aggregation of information and energy will become possible.

### 5.2. Microrobot working on a water surface

Apart from the approach in which rational structures are constructed at the 2D interface using lateral nanoarchitectonics to link functions, systems with functional autonomous objects propelled freely within the 2D surface are interesting as well. Candidates for such systems are nano- and microrobots that operate in an interfacial environment.^100^

**Fig. 19:**
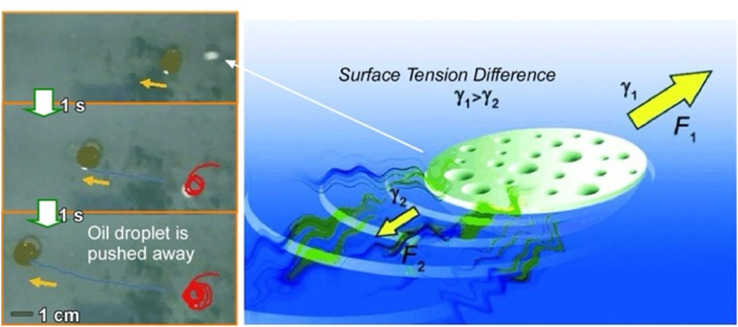
Polymer capsule motor running on a liquid surface upon asymmetric changes in the surface tension of the surrounding liquid due to the Marangoni effect. Photos on the left side demonstrate oil droplet pushing by the capsule motor. Reprinted with permission from ref. 101 Copyright 2011 Wiley-VCH.

The design and development of nano-, micro-, and millimeter-scaled autonomous systems capable of motion in an interfacial environment using various driving forces have attracted great attention. Pumera and co-workers developed a polymer capsule motor that was independent of external energy and studied its coordinated behaviour on the water surface (Fig. 19).^101^ The small millimeter-sized robot was propelled on the water surface without consuming any external energy or external fuel such as H_2_O_2_ or glucose. The driving force of the propulsion was an asymmetric release of organic solvent from the capsule. This resulted in an asymmetric change in the surface tension of the surrounding liquid. The molecular capsule motor moved toward the direction of high surface tension due to the Marangoni effect, trying to reach the desired lowest free energy state. The self-driven polymer capsule used here was fabricated by dropping a polysulfone solution in *N*,*N*′-dimethylformamide onto the surface of an aqueous liquid. When the polysulfone molecules interacted with water, they underwent a phase transition and solidified at the interface while forming small pores within the structure. *N*,*N*′-dimethylformamide was then slowly and asymmetrically released from the capsule to the solution/air interface through the pores, leading to self-propulsion abilities through a variety of liquid/air interfaces, including water, seawater, organic solvent/water mixtures, and acids. This property is useful in environmental applications. It is envisioned to clean up oil spills and toxic chemicals released into the environment.

**Fig. 20:**
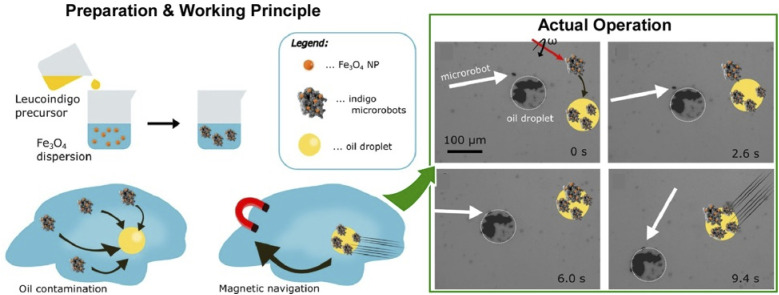
Magnetic navigation of indigo-based hydrophobic microrobots for oil removal: (left) preparation method and working principle; (right) actual operation for oil removal. Reprinted with permission from ref. 102 Copyright 2022 American Chemical Society.

Oil removal is undoubtedly a hot topic in environmental remediation. Jancik-Prochazkova *et al.* developed magnetically navigated indigo-based hydrophobic microrobots for oil removal (Fig. 20).^102^ The microrobots were fabricated by converting leucoindigo to insoluble indigo in the presence of commercially available spherical magnetic Fe_3_O_4_ nanoparticles. As a result of strong inter- and intramolecular hydrogen bonds and π–π interactions, indigo formed microparticles that were insoluble in water and common organic solvents. The presence of magnetic nanoparticles enabled wireless navigation in an external magnetic field. Moreover, a swarming behaviour was observed in a laterally rotating magnetic field. Due to their hydrophobic nature, the microrobots entered and subsequently transported oil contamination in a controlled way, enabling its removal from the aqueous environment.

In this section, we present some new challenges in research, mainly at the air–water interface. The Langmuir system is not all about the traditional approach of assembling and aligning lipid-like molecules. Liquid interfaces allow coordination chemistry and macromolecular chemistry to be deployed with restricted dimensions to create 2D COFs and MOFs. The incorporation of multiple components and step-by-step creation by employing the principles and tools of lateral nanoarchitectonics represents the next stage in the development of functional materials for a new era of electronic devices, solutions for environmental remediation, and beyond.

## 6. On aqueous membranes

In the previous sections, we considered solid interfaces with atomic resolution and air–water interfaces of clean monolayer-level thickness as suitable environments for developing lateral nanoarchitectonics. In this section, we take biological systems as a model for the lateral organization of functional molecules and their interactions. Therefore, we will focus on lateral nanoarchitectonics in lipid bilayers and membrane systems. In general, lipid bilayers and membranes are more dynamic units than those formed over solid surfaces or air–water interfaces because of their disordered structure. Their ability to immobilize functional elements, such as biomolecules, presents a very useful and interesting environment for functional coordination.^103^ In the following sections, we will discuss lateral nanoarchitectonics at aqueous membrane interfaces, including lipid bilayers.

### 6.1. DNA nanotubes and DNA origami

Media such as lipid bilayers have a high affinity for biological components, and the use of DNA aggregates as a mediator offers huge possibilities. DNA nanotechnology allows a high degree of design and assembly of complex structures.^104^ Examples of the application of DNA nanotubes and DNA origami to lateral nanoarchitectonics on membrane surfaces in an aqueous solution are presented below.

**Fig. 21:**
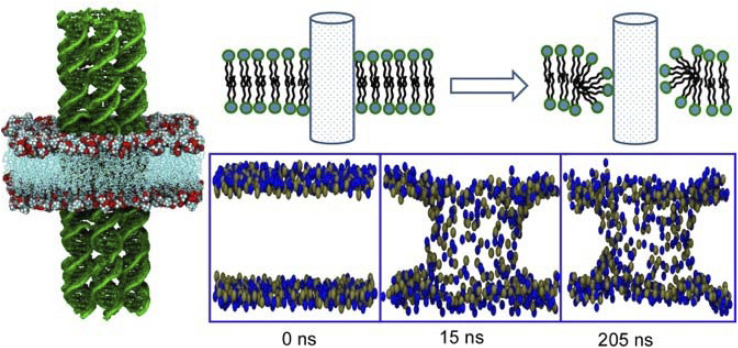
All-atom molecular dynamics simulation of tiled six-helical DNA nanotubes embedded in a 1-palmitoyl 2-oleoyl-*sn-glycero*-3-phosphocholine lipid bilayer where lipid molecules near the DNA nanotubes reorient to form a toroidal structure. Reprinted with permission from ref. 106 Copyright 2018 Oxford University Press.

A longstanding challenge in biotechnology is to rationally construct artificial channels in cell membrane models.^105^ The goal is to engineer synthetic nanopores that allow selective access to the interior of the cell through the lipid bilayer. One promising methodology is to embed DNA nanotubes into lipid bilayers. Joshi and Maiti analysed the stability and dynamics of six-helical tiled DNA nanotubes embedded in a 1-palmitoyl 2-oleoyl-*sn-glycero*-3-phosphocholine lipid bilayer in 0.2 μs long equilibrium.^106^ Lipid molecules in close proximity to DNA nanotubes reoriented to form a toroidal structure (Fig. 21). The head groups of lipid molecules near the membrane lumen cooperatively leaned toward the hydrophilic sugar–phosphate backbone of the DNA. As a result, the formation of a toroidal structure around the patch of DNA nanotubes protruding into the membrane was observed. This mechanism reduced the free energy barrier for the formation of the porous lumen of DNA nanotubes in the lipid bilayers. The energy barrier was eventually reduced by attaching a cholesterol anchor to DNA. The results of the all-atom molecular dynamics simulation performed in this study were useful for understanding the phenomenology of DNA nanotubes in lipid bilayers. This methodology sets important grounds for lateral nanoarchitectonics designs at membrane interfaces.

In ordinary supramolecular systems, it is challenging to control the long-distance movement of molecules. Cells, on the other hand, utilize molecular motors such as dynein and kinesin and cytoskeletons such as microtubules to move freely over long distances.^107^ Inspired by the molecular motors that enable intracellular transport, lateral nanoarchitectonics aims to mimic these systems by forming assemblies on membrane interfaces. Furuta and co-workers designed a protein motor that moved alongside DNA nanotubes.^108^ The motor was fabricated by combining dynein, a biomolecular motor, with a DNA-binding protein that was responsible for the selective motion on artificial DNA tracks with a precisely designed structure. In addition to this, the transport of multiple cargoes was presented by using different motors. This work introduced locally programmable and fully controlled molecular transport. The further development of peptides and other functional polymers and the contribution of lateral nanoarchitectonics will enable high-throughput production of new-era nano- and microdevices, such as external signal receivers, and systems for energy harvesting and information processing, among others.

**Fig. 22:**
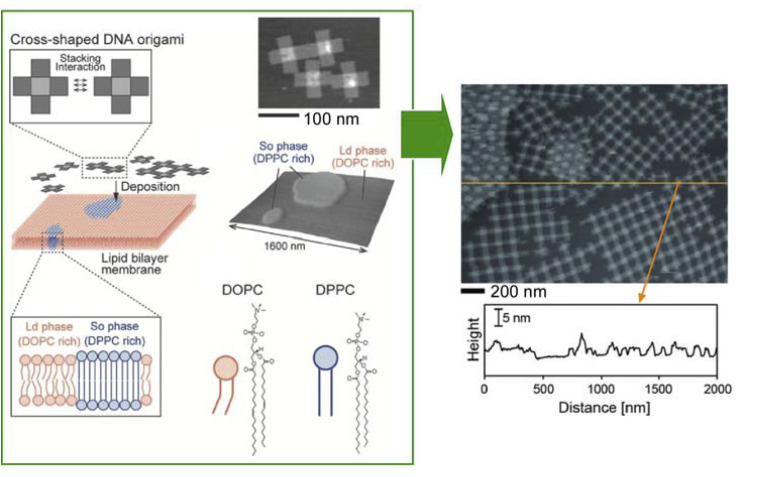
Environment-dependent self-assembly of DNA origami lattices on phase-separated lipid membranes: DNA origami on the liquid disordered phase formed 2D lattices, while DNA origami on the solid ordered phase formed aggregates (right AFM image of DNA origami on a lipid membrane). Reprinted with permission from ref. 109 Copyright 2018 Wiley-VCH.

Basic research on how to immobilize DNA origami on lipid membranes is crucial for the construction of advanced systems. Self-assembly of nanostructures such as DNA origami onto lipid membranes is an important element of lateral nanoarchitectonics. Suzuki and co-workers reported the environment-dependent self-assembly of DNA origami lattices on phase-separated lipid membranes.^109^ In particular, they investigated the environment-dependent assembly of DNA origami structures on phase-separated lipid bilayers consisting of a liquid disordered phase and a solid ordered phase (Fig. 22). The formation of 2D lattices depended on the fluidity of the lipids in the bilayer and on the charge density on the bilayer surface. At high charge density, DNA origami formed 2D lattices on the liquid disordered phase due to surface-mediated self-assembly. On the other hand, DNA origami in the solid ordered phase formed aggregates because of the lower mobility. There was also a significant influence of the ionic strength on the resulting 2D lattice. The presence of NaCl caused the lattices on the liquid disordered phase to desorb from the surface. In contrast, the DNA origami aggregates were reorganized into a lattice on the solid-order phase. In addition, the formation of the 2D lattice also depended on the lipid phase. As a result, it was possible to select the domain in which the lattice was formed. It was possible to design raft-like domains of DNA origami that responded to salt/thermal conditions. It can be assumed that combining functional DNA nanostructures by means of lateral nanoarchitectonics will lead to the fabrication of artificial cells and molecular robots.

**Fig. 23:**
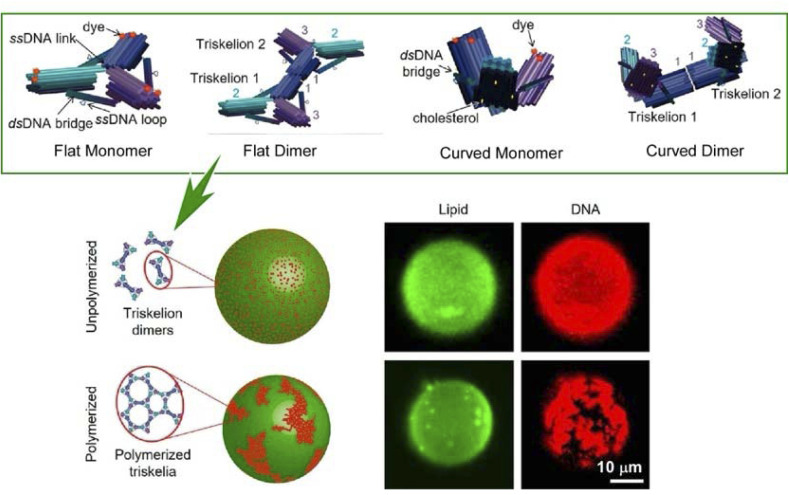
(Top) DNA triskelia in three-armed DNA origami nanostructures for dimers and different conformations; (bottom) polymerization of the triskelia on a lipid bilayer membrane to assemble into mesoscopic domains. Reproduced under terms of the CC-BY license from ref. 110, 2019 American Chemical Society.

The morphology of the membrane and its dynamic adaptations regulate many cellular functions. Advances in DNA nanotechnology have enabled DNA origami to adopt this role. DNA origami can be an effective tool to artificially control membrane morphology. Therefore, studying the interaction of DNA origami with lipid membranes during immobilization is also an important task in the field of nanoarchitectonics. Turberfield and co-workers examined the modification of membrane morphology and interactions with the formation of DNA origami clathrin mimic networks.^110^ In particular, they examined the assembly of DNA origami meshes on lipid membranes (Fig. 23). The original DNA triskelia were three-armed DNA origami nanostructures inspired by clathrin, a membrane-modifying protein. DNA origami was bound to lipid monolayers and lipid bilayers using cholesterol anchors; polymerization of the triskelia was triggered by the addition of DNA staples. As a result, the arms of the triskelia were linked to form a mesh. Large clusters of curved triskelia were formed as a result of polymerization. The unpolymerized DNA triskelia were uniformly distributed on the surface of the lipid bilayer vesicle. The network of polymerized triskelia caused submicrometer deformation of the lipid monolayer. This was like the formation of clathrin-coated pits. The polymerization of triskelia altered the interactions between the lipid bilayers. As a result, synapse formation between the giant unilamellar vesicles and the supporting lipid bilayer was inhibited. This study shows that the lateral nanoarchitectonics of DNA origami structures on the membrane surface provides a tool to control the dynamic behaviour of lipid membranes, their shape, and interactions. This will be useful for the development of biomimetic systems for signal transduction, synthesis, and reproduction based on membrane-bound compartments.

**Fig. 24:**
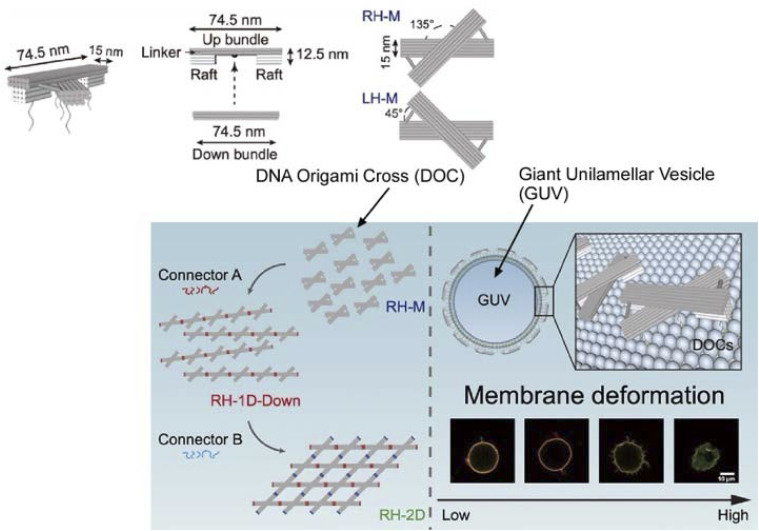
DNA origami cross structures anchored to giant unilamellar vesicles; polymerization of the DNA origami crosses deforms the membrane to different degrees depending on the degree of polymerization. Reprinted with permission from ref. 111 Copyright 2023 American Chemical Society.

The control of lipid membrane morphology by dynamic DNA origami networks was reported by Yang *et al.* (Fig. 24).^111^ In this study, DNA origami cross-structures were anchored to giant unilamellar vesicles. The adsorbed DNA origami crosses were polymerized into micrometer-scaled 1D chains or 2D lattices. The cross-chiral structure, which could be both polymerized and reconstituted, was demonstrated. Through this, the membrane morphology of the giant unilamellar vesicles was designed; polymerization after anchoring the DNA origami crosses to the giant unilamellar vesicles deformed the membrane depending on the degree of polymerization. To conclude, mimicking membrane deformation with programmable dynamic DNA nanostructures can mimic fundamental cellular processes such as endocytosis and exocytosis. When these findings are adopted, it is possible to develop DNA nanomachines that generate contractile forces and manipulate the morphology of living cells.

### 6.2. Artificial signal transduction

**Fig. 25:**
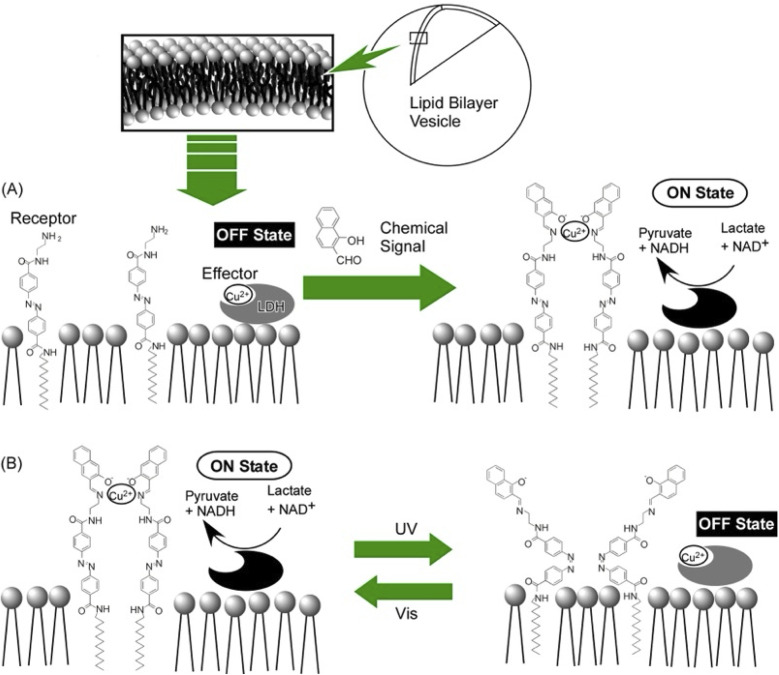
Artificial signal transduction with receptor molecules and lactate dehydrogenase (LDH) on lipid membranes: (A) chemical signal control and (B) photo signal control.

Cell membranes play a key role in signal transduction and photosynthesis. To perform such complex tasks, functional molecules, such as pigments and proteins, are rationally arranged within the cell membrane to perform highly efficient functions. Naturally, there have been several attempts to mimic similar systems. As shown below, an artificial receptor and an enzyme (lactate dehydrogenase, LDH) worked in tandem on a lipid bilayer to assemble an artificial signal transduction system.^112^ In this bilayer device, the artificial receptor was designed to initiate the enzymatic reaction after detecting an external chemical input signal (Fig. 25A). Here, LDH was immobilized in the lipid membrane with steroidal amines as effectors and receptors; the role of G proteins in the natural signal transduction system is carried out by copper ions, which are inhibitors of the enzyme. The mechanism of action was described as follows. First, the enzyme was inhibited (OFF state) in the presence of copper ions. When an aldehyde substance was introduced to the system, it bound to the artificial receptor upon the formation of a Schiff base that captured the copper ion inhibitors. With the inhibitor removed, the enzyme became active (ON state).

As a system in which the two signals are logically linked, an azobenzene-type receptor was used (Fig. 25B).^113^ When the azobenzene moiety was in the *cis* form, the recognition sites were not properly associated and the ability to capture copper ions was reduced. When the azobenzene moiety was in the *trans* configuration, the coordination ability of the copper ion was conformationally higher. Differences in enzyme activity were observed in systems containing *trans*- and *cis*-type receptors. At a concentration of 4 μM copper ions, the enzyme activity was 53% in the system containing the *trans*-type receptor, while it was only approximately 6% in the system with the *cis*-type receptor. The azobenzene-type receptor showed the same signalling behaviour as the steroidal-type receptor, meaning that exposure of LDH to copper ions for a long period caused certain irreversible changes and a decrease in LDH activity over time. Devices with the ability to repeatedly turn the activity of LDH on and off could be manufactured if appropriate conditions are chosen. The presented system can be thought of as a switching device with optical signals. It can also be thought of as a logic device in which two types of signals, chemical and optical, represent an input. The presence of a signalling molecule is considered true (absence is false), and irradiation with visible light is considered true (irradiation with ultraviolet light is false). In this system, the output, maintenance of enzyme activity, is obtained only when both the chemical and optical signals are true. Therefore, this system can be regarded as an AND-type logic circuit. Since this system can freely exchange and combine enzymes and receptors, it is expected that other types of logic circuits can also be developed. By combining several types of logic circuits, a nanothin-film calculator capable of simple arithmetic operations may be fabricated.

This can be achieved by using lipid bilayers as a medium to arrange functional components in lateral nanoarchitectonics to achieve functional linkage. Depending on the combination, switching and logic devices can be constructed. This concept has the potential to be deployed in a wide variety of ways.

### 6.3. Conjugated biomolecular machines

Similarly, there is also research on nanoarchitectonics of biomolecular machines and artificial structures on nanofilms prepared by lipid bilayer vesicles or LbL assembly to achieve interlocking functions. The research group led by Li and co-workers has made significant advances in this field.^114^ Some of the results are listed below.

Insight into biological pathways is crucial for designing and constructing organelle-like and cell-like structures. Lateral nanoarchitectonics represents a branch of the convergence of materials science and biodesign, enabling the construction of complex systems that mimic and enhance natural processes. Specifically, significant efforts have been made to improve energy conversion efficiency within biomimetic systems. Adenosine triphosphate (ATP), the primary energy currency of living organisms, plays an important role in the regulation of energy-dependent metabolic processes such as protein synthesis, signal transduction, and mass transport. Naturally, ATP is predominantly produced by ATP synthase in mitochondria, chloroplasts, and bacterial cytoplasmic membranes, driven by a transmembrane proton gradient. Lateral nanoarchitectonics for mimicking and modifying transmembrane-based subcellular functional units offers innovative approaches for bioenergy conversion.

**Fig. 26:**
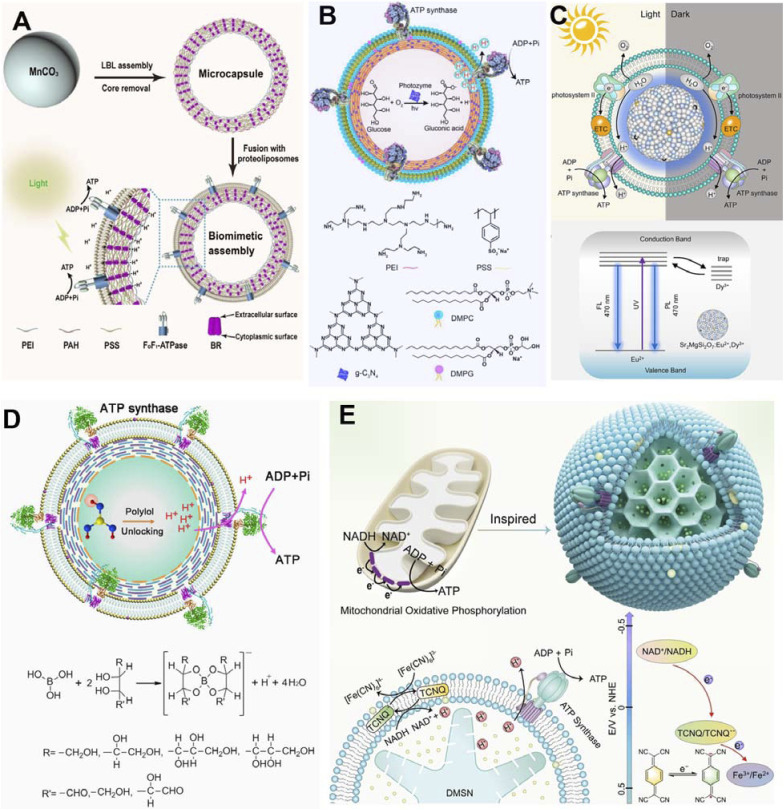
Schematics of various artificial bioenergy conversion systems. (A) LBL assembly of microcapsules using MnCO_3_ microspheres as removable templates and supporting FoF1-ATPase proteoliposomes. The enlarged view shows how oriented BR pumps protons integrated into the microcapsules, driving FoF1-ATPase to synthesize ATP from adenosine diphosphate (ADP) and inorganic phosphate (Pi) under light irradiation. (B) The ATP synthase-reconstituted architecture for light-driven oxidative phosphorylation. Under light irradiation, g-C_3_N_4_-PEI on the microcapsule shell acts as a photozyme, catalyzing glucose transformation into gluconic acid with oxygen, creating a proton gradient for ATP synthesis. (C) Biohybrid architecture integrating spectral and temporal light management. Based on the luminescence mechanism, under light irradiation, long afterglow particles (LAPs) can convert harmful UV light to visible light, enhancing the photosynthetic activity of natural thylakoid membranes (TM). In the dark, the long-life phosphorescence of LAPs can continue to drive photophosphorylation by TM. FL: fluorescence luminescence; PL: phosphorescence luminescence; ETC: electron transport chain. (D) An artificial bioenergy conversion system based on polyelectrolyte microcapsules supports ATP synthase-containing liposomes. In the presence of polyols, protons locked in boric acid are released, generating a proton gradient that drives ATP synthase to convert ADP and Pi into ATP. (E) An electron shuttle in an ATP synthase-reconstituted nanoarchitecture for enhanced bioenergy anabolism, inspired by natural mitochondria. The cross-section shows the bio-like system and transmembrane chemical reactions. Driven by tetracyanoquinodimethane (TCNQ) as the electron shuttle in the lipid bilayer, electrons flow from NADH to [Fe(CN)_6_]_3_. DMSM: dendritic mesoporous silica microparticle. Reprinted with permission from ref. 115 Copyright 2022 Wiley-VCH, ref. 116 Copyright 2023 American Chemical Society, ref. 117 Copyright 2023 Elsevier, ref. 118 Copyright 2021 Wiley-VCH, and ref. 119 Copyright 2024 Wiley-VCH.

A breakthrough developed by Li *et al.* involved improving ATP generation by constructing microcapsules with oriented bacteriorhodopsin (BR) using the LbL assembly technique (Fig. 26A).^115^ MnCO_3_ microspheres as removable templates, and polyetherimide (PEI), BR, poly(sodium-*p*-styrenesulfonate) (PSS), and poly(allylamine hydrochloride) (PAH) were successively adsorbed on the surface of the MnCO_3_ microspheres by LbL assembly. BR presented an oriented structure in the LbL assembly. To fabricate the microcapsules, the MnCO_3_ core was removed using ethylenediaminetetraacetic acid disodium salt (EDTA-Na_2_). The hollow microcapsules were then coated with FoF1-ATPase molecular motors. Oriented BR facilitated directional proton migration under illumination, thereby increasing the proton gradient necessary for ATP synthesis.

Another notable development involved the use of semiconducting graphitic carbon nitride (g-C_3_N_4_) nanosheets applied as a photozyme within microcapsules, co-assembled using the LbL deposition method with polyelectrolytes to mimic mitochondria (Fig. 26B).^116^ This system showed enhanced separation of photogenerated electron–hole pairs, accelerating the oxidation of glucose into gluconic acid and generating protons under light. These protons established an outward transmembrane proton gradient, which drove ATP synthase to synthesize ATP. This artificially designed assembly exhibited higher energy conversion efficiency than conventional oxidative phosphorylation systems, offering a novel method for chemical-to-biological energy conversion.

To further enhance photosynthetic efficiency, Li and co-workers co-assembled natural thylakoid membranes (TM) with artificial long afterglow particles (LAPs) (Fig. 26C).^117^ The LAP is known for its light conversion and storage capabilities. In this work, the LAP was optically matched with the absorption spectrum of TM. This assembly facilitated enhanced photosynthesis, as evidenced by increased rates of electron transfer, oxygen yield, and ATP production. Notably, the persistent phosphorescence emission from charged LAPs enabled continued photosynthesis in the dark, significantly improving natural systems that terminate photosynthesis immediately upon the onset of darkness.

The innovative approach of this group to ATP synthesis also included the use of boric acid as a new fuel source within an LbL assembly-related microcapsule (Fig. 26D).^118^ In this study, protons sequestered in boric acid were modulated and released by polyols. This controlled release of protons established a proton gradient across a lipid membrane, effectively driving the embedded ATP synthase to synthesize ATP from ADP and inorganic phosphate (Pi). This research introduced a novel method for bioenergy conversion and highlighted the potential of nonredox processes in artificial bioenergy production.

Lastly, to mimic the mitochondrial bioenergy anabolism, a nanoarchitecture was designed with a dendritic mesoporous silica microparticle (DMSM) as the inner compartment, which loaded NADH as the proton source and enabled rapid mass transfer (Fig. 26E).^119^ The outer compartment consisted of proteoliposomes reconstituted with ATP synthase. A synthetic electron shuttle, tetracyanoquinodimethane (TCNQ), embedded in the lipid bilayer, mediated transmembrane redox reactions, converting NADH to NAD^+^ and generating a proton gradient. This gradient led ATP synthase to rotate and synthesize ATP efficiently and sustainably, opening new avenues for enhanced bioenergy anabolism in a wide range of ATP-powered bioapplications.

### 6.4. Aqueous organic semiconductor films

The numerous examples in the previous sections have shown that nanoarchitectonics at the membrane interface in water is a useful approach to mimic biological functions and build advanced functional systems. Not only such biosystems, but nanoarchitectonics at the aqueous solution interface are also useful in a wide range of scientific and engineering fields. Finally, we discuss the importance of lateral nanoarchitectonics at the membrane interface using an example from a completely different field. This section will briefly introduce doping nanoarchitectonics for organic semiconductor thin films in conjunction with chemical equilibria at the aqueous interfaces.

**Fig. 27:**
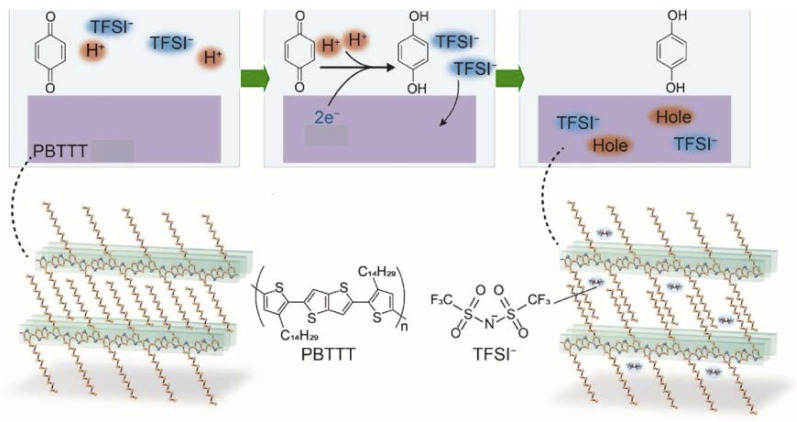
Doping of organic semiconductors attempted in an aqueous solution using benzoquinone and hydroquinone as proton-conjugated electron transfer reactions. Reprinted with permission from ref. 120 Copyright 2023 Springer-Nature.

Chemical doping using reactions with redox reagents was used to dope organic semiconductors. However, redox reagents are prone to degradation in the presence of water and/or air, and their use is restricted to environments of inert gases and vacuum. In addition, it is not easy to introduce dopant molecules without disturbing the crystal structure of organic semiconductors, which are formed only by weak intermolecular forces. Doping that meets all these challenges is not easy. Ishii, Yamashita and co-workers solved all these problems by molecularly doping nanoarchitectonics at the interface between aqueous solution and the thin film of organic semiconductors (Fig. 27).^120^ The inspiration came from coupling with the biochemical reaction, which takes place in water, *i.e.* proton-coupled electron transfer. It is a reaction in which positively charged protons and negatively charged electrons move in synchronisation with each other. Its equilibrium depends on the concentration of protons, also expressed as pH. Using this method, the redox reaction that occurs in water can be precisely controlled by using the pH of the aqueous solution. Doping of organic semiconductors was attempted in an aqueous solution using benzoquinone and hydroquinone as proton-conjugated electron transfer reactions. Thin films of organic semiconductors were immersed in aqueous solutions of benzoquinone, hydroquinone, and bis(trifluoromethylsulfonyl)imide anion (TFSI^−^). The TFSI^−^ represented an anion that plays the role of a dopant in p-type semiconductors. The benzoquinone used in this study was converted to hydroquinone without producing an anion molecule in the process. Therefore, the hydrophobic anion TFSI^−^ was additionally dissolved in the dopant solution. When benzoquinone was oxidized a hole was formed in the organic semiconductor film, enabling the incorporation of the TFSI^−^ dopant. This division of tasks enabled the use of proton-conjugated electron-transfer-type redox reagents such as benzoquinone/hydroquinone for chemical doping. A notable advantage of using benzoquinone/hydroquinone solutions is their excellent reproducibility and pH-dependent controllability. By adjusting the pH of the aqueous solution, the electrical conductivity of the organic semiconductors was controlled with high precision. This doping nanoarchitectonics is an extremely simple process that involves only immersion of a thin film of organic semiconductors in an aqueous solution. When the process is performed in water, the method is able to overcome the traditional problems of organic semiconductor doping. These processes are based on coupling of chemical equilibrium and membrane lateral electrical conductions at the interface. The construction of membrane systems by lateral nanoarchitectonics on such doping systems would create circuit-like two-dimensional architectures.

In several of the previous sections, we have presented diverse examples of lateral nanoarchitectonics at membrane interfaces in contact with aqueous solutions. Operating in aqueous solution systems and using lipid bilayer structures as interfacial media, a wide variety of biomaterials can be assembled for functionality; elements that can be highly programmable in structure can be incorporated, such as DNA origami, enzymes, and biomolecular motors. It was also demonstrated that finding inspiration in biological equilibrium reactions allows for precise doping nanoarchitectonics of organic semiconductors. In lateral nanoarchitectonics at the aqueous interface, advanced biological phenomena and semiconductor engineering can be coupled. This suggests a promising field for the development of new innovative functions, such as wet devices.

## 7. Living cells at the liquid–liquid interface

Many of the previous examples have focused on functional coordination in various types of cellular membranes. Cellular membranes comprise highly organized molecules that allow effective signal transmission to the interior of the cell and direct various complex biological functions. The plasma membrane, as well as other organelle membranes, is therefore an ideal platform for developing lateral nanoarchitectonics. However, lateral nanoarchitectonics is not limited to mimicking and modifying the plasma membrane or organelle membranes to transport signals or enhance biological energy conversion. It can also be a crucial approach for the construction of the extracellular matrix (ECM) and the fine-tuning of the regulation of cellular fate. To fully understand the potential applications of lateral nanoarchitectonics, it is important to examine how cells interact with artificial interfaces. Although there is extensive research on cell behaviour on solid surfaces,^121^ the study of cell behaviour at liquid–liquid interfaces—independent of solid environments—is still in the early stages.^122^

Some notable advancements in this area are highlighted in the following section. For example, Jia and colleagues have presented significantly advanced lateral nanoarchitectonics for ECM confinement in cell culture, based on perfluorocarbon–water interfacial systems. This research underscores the importance of exploring how mechanical cues in cellular microenvironments influence key cell functions, such as spreading and differentiation.

**Fig. 28:**
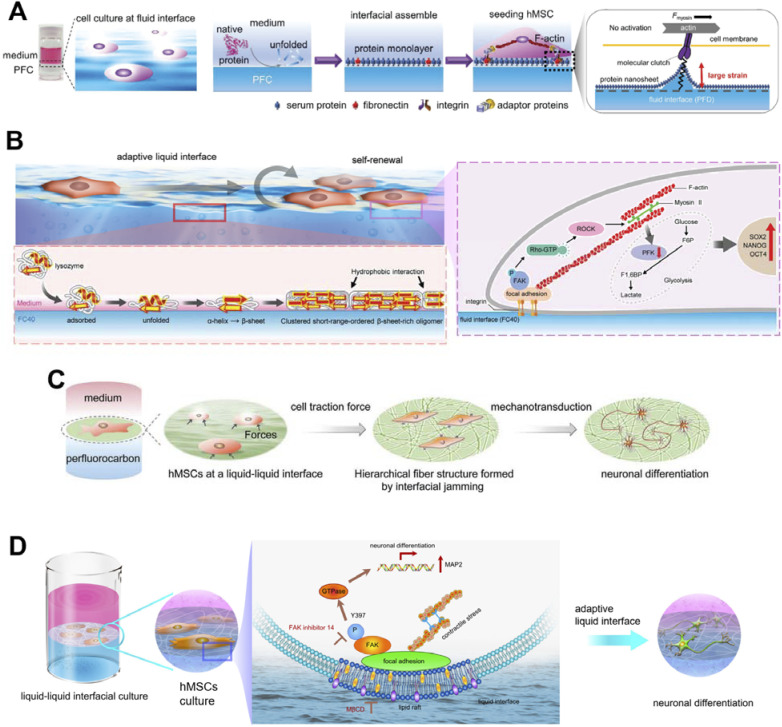
Liquid–liquid interfacial assembled 2D protein monolayer nanosheet based culture system for hMSC fate controlling. (A) A photograph and illustrations present the formation of a protein monolayer at the liquid–liquid interface, resulting from the denaturation and self-assembly of serum proteins. The response of hMSCs to the stiffness of the protein monolayer is clarified through the actin–integrin–fibronectin (FN) clutch mechanism. (B) Lysozyme monolayers, self-assembled at the liquid interface, are shown to maintain the self-renewal and multipotency of hMSCs. The lysozyme forms a close-packed monolayer through hydrophobic interactions among β-sheet-rich oligomers. Enhancing the retention of multipotency by reducing cytoskeletal tension and glycolysis activity was verified. The figure also labels key glycolytic intermediates: fructose 6-phosphate (F6P) and fructose 1,6-bisphosphate (F1,6BP). (C) The adaptive self-assembled protein monolayer is responsive to the traction forces produced by cells, which in turn guides the differentiation of hMSCs. (D) The figure illustrates the neuronal differentiation of hMSCs at the interface, where a 2D network of protein nanofibrils is assembled. This differentiation process is related to lipid raft assembly and the phosphorylation of focal adhesion kinase (FAK). Reprinted with permission from ref. 123 Copyright 2019 Wiley-VCH, ref. 124 Copyright 2023 Wiley-VCH, and ref. 125 Copyright 2019 Wiley-VCH. Reproduced under terms of the CC-BY license from ref. 126, 2022 Springer-Nature.

Traditionally, studies used solid substrates, assuming cells cannot spread on fluid substrates due to rapid relaxation, which fails to resist actomyosin-based cell contractility. However, Jia *et al.* demonstrated that anchorage-dependent cells, including human mesenchymal stem cells (hMSCs), can spread and grow at the liquid interface between a perfluorocarbon fluid and the culture medium (Fig. 28A).^123^ This phenomenon was facilitated by the self-assembly of a monomolecular protein nanosheet at the fluid interface, which provided sufficient rigidity to support cell spreading without additional treatment. The stiffness of the protein monolayer was regulated by fine-tuning the packing of proteins at the liquid interface. The increased stiffness of the protein nanosheets triggered cell spread, adhesion growth, and nuclear translocation of the yes-associated protein (YAP). This behaviour aligned with the molecular clutch model, offering insights into how cells interact with such adaptive fluid environments. Furthermore, these freestanding ultrathin protein nanosheets were extremely flexible, easily deformed, and perceived by cells as much softer than solid substrates, providing new perspectives on cell–material interactions.

The dynamic nature of the native ECM involves continuous feedback between cells, which plays a crucial role in regulating cell functions. A lysozyme molecule lacks specific integrin-binding motifs; however, lysozyme-assembled nanosheets present naturally positively charged surfaces that facilitate cell attachment and the subsequent spread of cells. Lyu *et al.* built an adaptive environment based on self-assembled lysozyme monolayers at the FC40 perfluorocarbon–water interface that attempted to mimic the dynamic nature of the ECM (Fig. 28B).^124^ The dynamic adaptivity of interfacial assembled protein nanosheets was modulated independent of bulk mechanical properties through covalent crosslinking, allowing bidirectional interactions between cells and liquid interfaces of varying dynamic adaptivity. This approach enhanced the growth and multipotency of hMSCs, mediated by low cell contractility and metabolic activity, involving continuous mutual feedback between cells and materials.

Adaptive materials composed of a protein monolayer assembled at a liquid–liquid interface dynamically adapt to cell traction forces. Jia *et al.* investigated detailed cell–material interactions by establishing a fibronectin-enriched protein monolayer at the perfluorocarbon–water interface for stem cell culture. Through interfacial jamming, an ultrastructure transition from a protein monolayer to hierarchical fibers was reported to promote the kinase activation and neuronal differentiation of stem cells (Fig. 28C).^125^ Cell traction forces resulted in the spatial rearrangement of ECM proteins, which reacted to alter the stem cell fate. This biomimetic adaptive liquid interface enabled dynamic control of stem cell behaviour, with significant translational potential.

It is understood that the fates of stem cells are cooperatively driven by their microenvironment interactions. Biomaterials are dynamically remodelled by stem cells, which sense and translate these changes into cell fate decisions. Previously reported adaptive biomaterials composed of fibronectin inserted into protein nanosheets at a liquid interface enhanced neuronal differentiation of hMSCs. However, distinguishing the effects of ligand density from those of fibrillary structure on cellular function and fate was challenging. Jia *et al.* constructed a novel adaptive biomaterial using 2D networks of protein nanofibrils assembled at a liquid–liquid interface (Fig. 28D).^126^ Compared to flat protein nanosheets, 2D hierarchical nanosheets based on protein nanofibrils improved the neuronal differentiation of hMSCs through a focal adhesion kinase signalling mechanism. The lipid raft microdomains in the plasma membrane played a central role in how the hMSCs rapidly adapted to the dynamic microenvironment at the fluid interface. These findings have substantial implications for regenerative medicine and tissue engineering, highlighting the importance of individualized and precise design adaptive biomaterials in controlling cell behavior and fate.

Inspired by adaptive mechanisms found in natural systems, lateral nanoarchitectonics has paved the way for the creation of reconfigurable all-liquid structures, offering a unique approach to material design. These systems, where living cells are anchored at liquid–liquid interfaces, provide a soft, flexible, and dynamic platform that adapts to mechanical forces generated by the cells themselves. This dynamic environment, which closely mimics the natural extracellular matrix, supports cell growth, signal transmission, and various complex biological functions in real time.

One of the most promising applications of this technology is in the field of stem cell research. Stem cells, due to their multipotency, can differentiate into various cell types, including bone, cartilage, and neural cells. By utilizing lateral nanoarchitectonics at liquid–liquid interfaces, a controlled environment is created to fine-tune the biochemical and biophysical cues necessary for directing stem cell fate. For example, softer, more elastic surfaces may favor neural cell development, while bioactive molecules introduced at the interface can enhance precision, creating highly specialized cellular environments tailored for tissue-specific engineering.

The ability to control stem cell behavior in this way is particularly significant for regenerative medicine, where precise differentiation is crucial for replacing damaged tissues with functional ones. Platforms designed using lateral nanoarchitectonics at the perfluorocarbon–water interface not only support cell growth but also guide it in a highly controlled manner, opening up new possibilities for creating tissues that closely resemble their natural counterparts in both structure and function. This could greatly improve treatments for conditions such as bone fractures, cartilage damage, and neurodegenerative diseases.

Overall, lateral nanoarchitectonics holds great potential for advancing biomedical science by leveraging the unique properties of liquid–liquid interfaces and integrating functional nanoscale materials. This approach positions nanoarchitectonics as a key technology in the development of next-generation medical treatments, where biomaterials interact with cells in adaptive and responsive ways.

## 8. Summary and perspectives

In this review paper, under the theme of lateral nanoarchitectonics, we have examined the rational extension of functional structures within a single plane: their organization, interlocking of functional units, and the exploration of the potential for the expression of advanced bioinspired functions. Overall trends have been explored, encompassing a wide variety of examples that range from the creation of structures at the molecular level to the regulation of cell differentiation. In many systems, we found that, while the targets are very attractive, the research is in its infancy, and many challenges remain to be overcome.

Molecular lateral nanoarchitectonics is in its early stages of research in terms of structural complexity and function. The extension of molecular structures by on-surface synthesis is in its infancy, but is conceptually highly innovative. These efforts break the rules of organic synthesis and change the history of organic chemistry. The organic synthesis pathway of on-surface synthesis is not based on probabilistic collisions or energy stability in solution. Conventional molecular synthesis is limited by the rules of organic chemistry. Lateral nanoarchitectonics has the potential to develop completely new trends in organic chemistry by inserting targeted functional groups into targeted sites on a plane. Although some work needs to be done in terms of productivity, the use of lateral nanoarchitectonics in nano-level devices may lead to a revolutionary development. The interlocking of molecular gears also provides an example of something that is imaginatively possible, but difficult to realize in practice. However, once the fundamentals of molecular machine manipulation are established at a given position on a surface, the stage of building it will proceed quickly. Such an example was achieved in surface-operated systems with DNA origami. Supramolecular coordination of molecular machines on surfaces may lead to the construction of smaller, more precise, but intricately interlocking machines on surfaces.

Many lateral nanoarchitectonics systems that incorporate biofunctional molecules are functionally advanced. It is meaningful to organize molecular motors, enzymes, and other molecules that themselves exhibit high functionality on membranes in terms of functional coordination. Although actual functional coordination has been achieved, there are few examples of advanced functional systems in which many functional elements work in tandem. More efforts should be made to artificially create functional systems such as those found in cells in which a number of functional systems work in tandem upon stimulation from the interface. Lipid membrane raft models and DNA origami on lipid membranes can be used as tools for this purpose. Although not mentioned in this review, peptide assembly structures have also been used to reproduce advanced biological functions. Programmed peptide assembly structures^127^ as well as much advanced DNA nanotechnologies may also be useful for such functional organization. It is hoped that more advanced functional systems containing biological functions will be constructed by lateral nanoarchitectonics using these tools.

Lateral nanoarchitectonics, in which more complex recognition sites are formed by the assembly of simple molecules in a single molecular plane at the water surface, is a pioneering concept. However, this field does not seem to be developing rapidly. New developments seem to require integration with the synthesis of MOFs and COFs at interfaces and progress in the synthesis of supramolecular polymers and gel fibers. These technologies are often based on single or simple components. With the development of techniques such as multicomponent structure formation and stepwise structure evolution, more complex structures can be designed in the lateral direction within the secondary exemptions. In such a case, if a system such as receptor creation by molecular assembly is incorporated, it will be possible to construct a kind of cascade system in which multiple functions are interlocked, or a 2D energy-intensive functional system. Lateral nanoarchitectonics, such as 2D dendrimer-like MOFs, COFs, and supramolecular polymers, may be required. As a dynamic linkage element, the use of micro-robots that run on their own at the interface is also attractive. The creation of bio-/artificial devices by linking such interlocking functions with organic semiconductor thin films also represents a futuristic challenge.

A specific and unique system in nanoarchitectonics at the interface is the coupling of macro- and nanoscale phenomena. Molecular machines arranged at the interface can be subjected to macroscopic mechanical stimuli from the lateral direction of the membrane. The collective motion of molecular rotors at the liquid interface can also be detected as macroscopic signals. This is because the liquid interface can link the macroscopic motion in the lateral direction with the molecular motion on the membrane surface. So far, such an idea has only been used for simple molecular machine systems, and when more sophisticated functional coordination systems are constructed in the interfacial membrane by lateral nanoarchitectonics, they will be controllable by macroscopic motion. Macroscopic motion is compatible with the movements of human daily life. Therefore, if such a 2D complex system is constructed, it will be possible to control advanced functional systems with human daily movements.

Of course, all the possible candidates in lateral nanoarchitectonics cannot be mentioned in one review article. We have to note that there are many other possibilities. To complement this molecular machine's intricate mechanical design, Dip-Pen Nanolithography (DPN) offers an alternative method for molecular manipulation, focusing not on interlocking motions but on precise molecular deposition and surface patterning.^128^ This lateral organization of molecules on surfaces is a core aspect of lateral nanoarchitectonics, enabling the construction of nanoscale devices where surface interactions and patterning are critical. By precisely controlling molecular deposition, DPN allows for the development of highly specialized materials and structures, offering new possibilities in areas such as molecular electronics, biosensing, and nanofabrication. Similar emphasis has to given to the other powerful candidates. Supported bilayer membranes^129^ definitely have important contributions to lateral nanoarchitectonics.

However, there is a danger of falling into a too diverse and individualistic discussion. Therefore, a data-driven approach is important rather than relying on the experience and knowledge of researchers. It is worth noting that artificial intelligence is making remarkable progress and machine learning enables optimization and direct research in a data-driven manner.^130^ In fact, in the development of nanoporous materials, the necessity of integrating material informatics and nanoarchitectonics has been discussed.^131^ The development of functional systems by lateral nanoarchitectonics will also be advanced to more complex and sophisticated systems by the introduction of artificial intelligence.

## Data availability

No primary research results, software or code have been included and no new data were generated or analysed as part of this review.

## Author contributions

J. S.: conceptualization, writing, review & editing. A. J.-P.: conceptualization, writing & editing. K. K.: project administration, review & editing. K. A.: conceptualization, writing, review & editing, funding acquisition.

## Conflicts of interest

There are no conflicts to declare.
